# METTL3 regulates autophagy of hypoxia-induced cardiomyocytes by targeting ATG7

**DOI:** 10.1038/s41420-025-02320-3

**Published:** 2025-02-01

**Authors:** Linnan Li, Hao Cheng, Yufei Zhou, Di Zhao, Xiaoxue Zhang, Yajun Wang, Jianying Ma, Junbo Ge

**Affiliations:** 1 Department of Cardiology, Zhongshan Hospital, Shanghai Institute of Cardiovascular Diseases, Fudan University, Shanghai, China; 2 State Key Laboratory of Cardiology, Zhongshan Hospital, Fudan University, Shanghai, China; 3 Key Laboratory of Viral Heart Diseases, National Health Commission, Shanghai, China; 4 Key Laboratory of Viral Heart Diseases, Chinese Academy of Medical Sciences, Shanghai, China; 5 National Clinical Research Center for Interventional Medicine, Shanghai, China; 6 Institutes of Biomedical Sciences, Fudan University, Shanghai, China; 7 Department of Pulmonary and Critical Care Medicine, Zhongshan Hospital, Fudan University, Shanghai, China

**Keywords:** Macroautophagy, Myocardial infarction

## Abstract

N^6^-methyladenosine (m^6^A) mRNA modification is the most common mRNA internal modification in eukaryotes, which participates in a variety of biological processes. However, the role of m^6^A methylation in regulating autophagy induced by ischemia and hypoxia remains to be widely investigated. Here, we investigated the impact of METTL3, a key m^6^A methyltransferase, on the autophagy regulation in ischemic and hypoxic cardiomyocytes, as well as in mice following acute myocardial infarction (AMI). METTL3 negatively regulated autophagy in cardiomyocytes under ischemia and hypoxia conditions. Silencing METTL3 enhanced autophagy and mitigated cardiomyocyte injury, whereas overexpression of METTL3 exerted the opposite effect. Mechanistically, METTL3 methylated ATG7 mRNA, a crucial autophagy-related gene, leads to the recruitment of the m^6^A-binding protein YTHDF2. Subsequently, YTHDF2 facilitated the degradation of ATG7 mRNA, consequently inhibiting autophagy and exacerbating cellular damage. Our study shed light on the pivotal role of METTL3-mediated m^6^A modification in the regulation of autophagy during AMI, providing novel insights into the functional significance of m^6^A methylation and its regulatory mechanisms.

## Introduction

Acute myocardial infarction (AMI) is a prevalent cardiovascular disease characterized by ischemic cardiomyocyte death and is a leading cause of global mortality [1]. Despite advancements in drug and interventional therapies, effective treatments are still lacking, underscoring the importance of uncovering novel mechanistic insights into AMI pathogenesis for the development of preventive and therapeutic strategies.

Epigenetics refers to the regulation of gene expression without changing the DNA sequence [2], including reversible modifications at DNA, histone, and RNA levels [3]. Among these, m^6^A RNA methylation is the most common methylation modification in the internal sequence of eukaryotic mRNA [4, 5]. It mainly regulates protein expression by regulating mRNA stability, splicing, nuclear transport, translation, and degradation [6].

In the cellular environment, m^6^A methylation is regulated by three protein groups, including methyltransferase (writers), m^6^A binding protein (readers,) and demethylase (erasers) [7, 8]. Among them, METTL3, a key component of the methyltransferase complex, exhibits fundamental catalytic activity as an N6-methylase and has been extensively studied [9, 10]. Previous research has reported that METTL3 was necessary for normal hypertrophy of cardiomyocytes. METTL3-mediated methylation enhancement led to compensatory cardiac hypertrophy, while reduced m^6^A led to eccentric cardiomyocyte remodeling and dysfunction [11]. Additionally, the reduction of m^6^A mediated by METTL3 deletion inhibited the maturation of pri-miR-143 and promoted the proliferation of newborn cardiomyocytes at least partly by targeting Yap and Ctnnd1 [12]. Therefore, m^6^A methylation mediated by METTL3 is closely related to cardiovascular diseases. Although the relationship between METTL3 and AMI has been reported, the underlying mechanism remains unclear and requires further investigation.

Autophagy, an evolutionarily conservative mechanism, exists widely in eukaryotes [13]. In humans, baseline autophagy is essential for cell growth, survival, and homeostasis, while dysregulated autophagy can contribute to various pathophysiological conditions, including cancer, cardiovascular disease, aging, neurodegenerative disease, and so on [14]. Macroautophagy, the most common form of autophagy, has been shown to be influenced by epigenetic modifications, particularly m^6^A RNA methylation [15]. m^6^A RNA modification can directly affect the expression of autophagy-related genes and regulate autophagy [16]. Recent research has revealed that METTL3-mediated m^6^A modification increased the stability of TFEB transcripts, promoting autophagy and inhibiting apoptosis of cardiomyocytes treated with hypoxia-reoxygenation [17]. Meanwhile, METTL3 could also inhibit the phenotypic transformation of vascular smooth muscle cells by positively regulating the formation of autophagosomes mediated by ATG5 and ATG7 [18]. However, the regulatory mechanisms of METTL3 on ischemic and hypoxic cardiomyocyte autophagy in the context of AMI remain elusive.

Therefore, this study aims to investigate the role of METTL3-mediated m^6^A RNA methylation modification in the autophagy of ischemic and hypoxic cardiomyocytes, and explore the mechanism of m^6^A modification involved in ischemic heart diseases.

## Results

### METTL3 regulated autophagy in hypoxia-induced cardiomyocytes

**Fig. 1:**
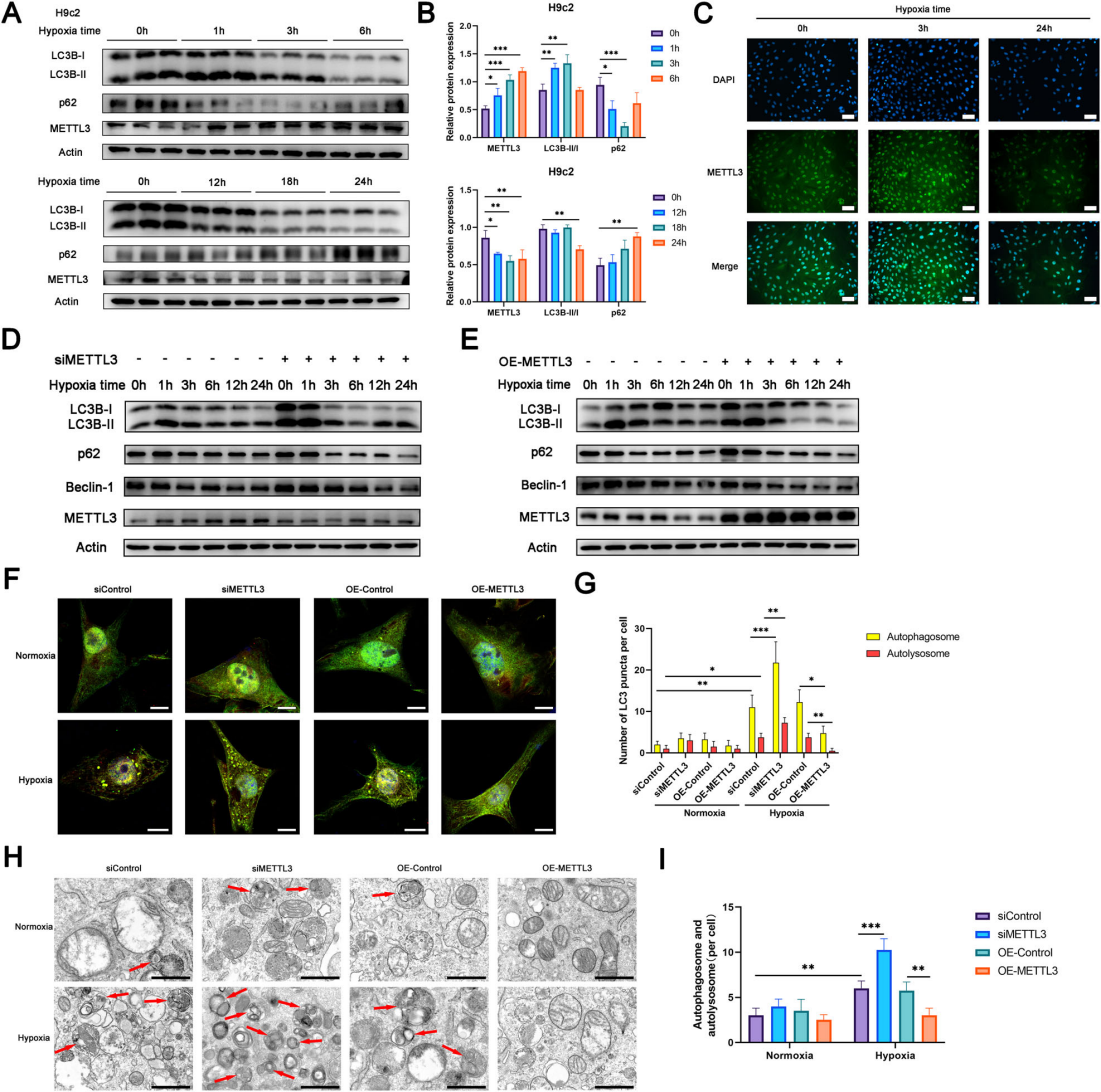
METTL3 regulated autophagy in hypoxia-induced cardiomyocytes. **A**, **B** H9c2 cells were exposed to hypoxia for 0 h, 1 h, 3 h, 6 h, 12 h, 18 h, and 24 h, respectively, and the protein levels were analyzed by Western blotting (*n* = 3). **C** The representative images of immunofluorescence staining for METTL3 in H9c2 cells with or without hypoxia (*n* = 3). Scale bar: 100 μm. **D** Western blot analysis of protein levels in H9c2 cells with METTL3 knockdown following hypoxia for the indicated time periods (*n* = 3). **E** Western blot analysis of protein levels in H9c2 cells with METTL3 overexpression following hypoxia for the indicated time periods (*n* = 3). **F**, **G** EGFP-mCherry-LC3 was transfected into NRCMs, and autophagosome (yellow) and autolysosome (red) formation was examined in METTL3 knockdown or METTL3 overexpressing cells, with or without hypoxia (*n* = 5). Scale bar: 10 μm. **H**, **I** Transmission electron microscopy analysis of autophagosomes and autolysosomes in METTL3 knockdown or METTL3 overexpressing cells, with or without hypoxia (*n* = 5). Arrows indicate autophagosomes and autolysosomes. Scale bar: 1 μm. All data were presented as mean ± SD. ns represents *p* > 0.05, ^*^p < 0.05, ^**^p < 0.01, ^***^p < 0.001, ^****^p < 0.0001.

Autophagy is considered a therapeutic target for ischemic heart disease and can occur post-myocardial infarction [19]. To elucidate the connection between METTL3 and autophagy, we analyzed the changes of METTL3 and autophagy-related proteins in H9c2 cells (Fig. 1A, B) and NRCMs (Fig. S1A, B) under different durations of hypoxia. After treatment with a hypoxic solution and exposure to a 1% O_2_ hypoxic environment to simulate myocardial infarction, Western blot analysis revealed that the levels of METTL3 and autophagy initially increased and then decreased with prolonged hypoxia time. Specifically, our study found that autophagy levels peaked in H9c2 cells after 3 h of hypoxia and in primary cardiomyocytes after 6 h of hypoxia, with significant autophagy occurring early in both cells. In addition, the expression of METTL3 was also significantly altered under both time points. Therefore, we ultimately set the hypoxia duration to 3 h for H9c2 cells and 6 h for NRCM cells, and all subsequent hypoxia experiments performed under these conditions. Immunofluorescence staining also showed the expression of METTL3 during hypoxia, while METTL3 was predominantly localized in the nucleus regardless of the duration of hypoxia (Fig. 1C).

To investigate the effect of METTL3 on autophagy in ischemic and hypoxic cardiomyocytes, H9c2 cells were divided into two groups: a control group and a METTL3 knockdown group. After hypoxia, we measured the protein levels of MAP1LC3B/LC3, SQSTM1/p62, and Beclin-1 to determine the level of autophagy (Fig. 1D). We found that METTL3 knockdown increased the ratio of LC3B-II/LC3B-I and the expression of Beclin-1, while decreasing the level of SQSTM1. Conversely, METTL3 overexpression significantly decreased the ratio of LC3B-II/LC3B-I and the expression of Beclin-1, while increasing the expression of SQSTM1 (Fig. 1E), which indicated that METTL3 was negatively correlated with autophagy. In addition, immunofluorescence analysis showed that METTL3 overexpression significantly decreased the formation of LC3 spots in H9c2 cells, while METTL3 knockdown increased the formation of LC3 spots (Fig. 1F, G). Subsequently, we used transmission electron microscopy to analyze the formation of autophagosomes in each group, and we found that METTL3 overexpression reduced the number of autophagosomes, indicating inhibited autophagy, while interfering with METTL3 increased the number of autophagosomes in H9c2 cells (Fig. 1H, I).

Consistent with H9c2 cells, overexpression and knockdown of METTL3 in NRCMs also weakened and enhanced autophagy, respectively (Fig. S1C, D). Similarly, METTL3 knockdown in other myocardial cell lines, such as AC16, also enhanced autophagy (Fig. S1E). These results collectively indicated that METTL3 negatively regulated autophagy activation in cardiomyocytes.

### METTL3 affected the injury of hypoxic cardiomyocytes by autophagy

**Fig. 2:**
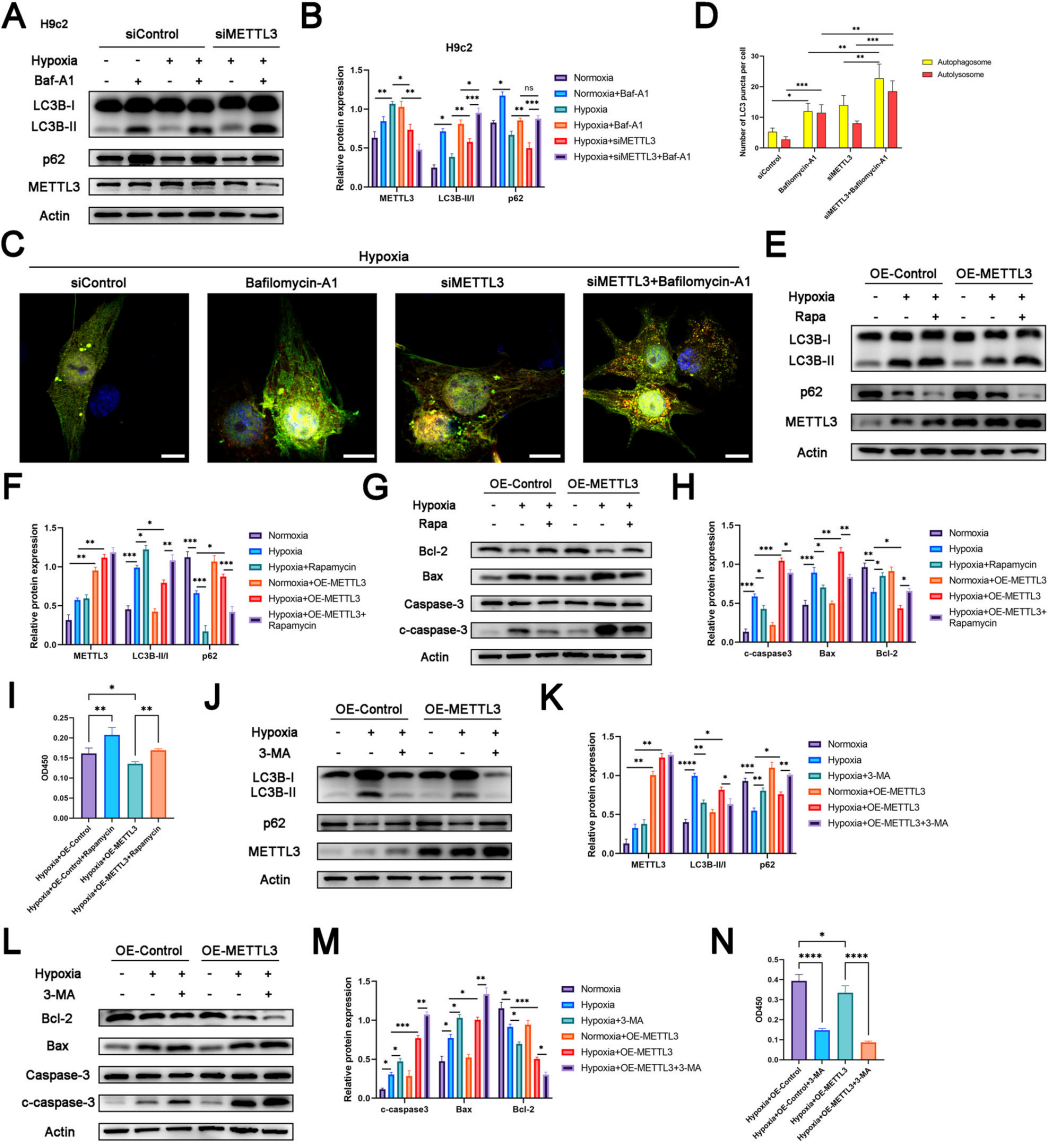
METTL3 affected the injury of hypoxic cardiomyocytes by autophagy. **A**, **B** Western blot analysis of protein levels in control and METTL3 knockdown H9c2 cells. The cells were treated with or without Bafilomycin A1 (BafA1, 100 nM) for 3 h prior to incubation with or without hypoxia (*n* = 3). **C**, **D** EGFP-mCherry-LC3 was transfected into NRCMs, and autophagosome (yellow) and autolysosome (red) formation was examined in control and METTL3 knockdown cells treated with or without Bafilomycin A1 (BafA1, 100 nM) for 3 h (*n* = 5). Scale bar: 10 μm. **E**, **F** Western blot analysis of protein levels in control and METTL3 overexpressing cells. The cells were treated with or without Rapamycin (Rapa, 500 nM) for 24 h prior to incubation with hypoxia (*n* = 3). **G**, **H** Western blot analysis of protein levels in control and METTL3 overexpressing cells. The cells were treated with or without Rapamycin (Rapa, 500 nM) for 24 h prior to incubation with hypoxia (*n* = 3). **I** Cell viability was evaluated with the CCK8 assay (*n* = 5). **J**, **K** Western blot analysis of protein levels in control and METTL3 overexpressing cells. The cells were treated with or without 3-methyladenine (3-MA, 5 mM) for 12 h prior to incubation with hypoxia (*n* = 3). **L**, **M** Western blot analysis of protein levels in control and METTL3 overexpressing cells. The cells were treated with or without 3-methyladenine (3-MA, 5 mM) for 12 h prior to incubation with hypoxia (*n* = 3). **N** Cell viability was evaluated with the CCK8 assay (*n* = 5). All data were presented as mean ± SD. ns represents *p* > 0.05, ^*^p < 0.05, ^**^p < 0.01, ^***^p < 0.001, ^****^p < 0.0001.

Autophagy encompasses a multi-step biological process [20]. To observe the effect of METTL3 on autophagy flux in ischemic and hypoxic cardiomyocytes, we used autophagy inhibitor Bafilomycin-A1 (a late autophagy inhibitor) to treat H9c2 cells (Fig. 2A, B) and NRCMs (Fig. S2A, B). The results showed that METTL3 knockdown induced autophagy at least by promoting the occurrence and development of autophagosomes in the early stage of autophagy. In addition, we used mCherry-EGFP-LC3 adenovirus to transfect cardiomyocytes for immunofluorescence analysis, and found that although Bafilomycin-A1 inhibited the late autophagic pathway in cells, the yellow granules (representing autophagosomes) and red granules (representing autolysosomes) in METTL3 knockdown cells increased significantly (Fig. 2C, D), indicating that METTL3 affected the early stages of autophagy in ischemic and hypoxic cardiomyocytes.

To further clarify whether METTL3 plays a key role in the injury of ischemic and hypoxic cardiomyocytes by regulating autophagy, we used autophagy tools including Rapamycin (an autophagy agonist) and 3-MA (an autophagy inhibitor) to treat H9c2 cells. In both the control group and METTL3 overexpression group, the autophagy flux significantly increased when the autophagy agonist Rapamycin was added, resulting in increased cardiomyocyte viability and decreased apoptosis levels (Fig. 2E–I). In contrast, treatment with the autophagy inhibitor 3-MA significantly reduced autophagy flux, resulting in the opposite effect on cardiomyocyte viability and apoptosis level (Fig. 2J–N). These data suggested that METTL3 knockdown protected cardiomyocytes from hypoxia-induced injury by promoting autophagy flux.

### METTL3 affected autophagy by targeting ATG7

**Fig. 3:**
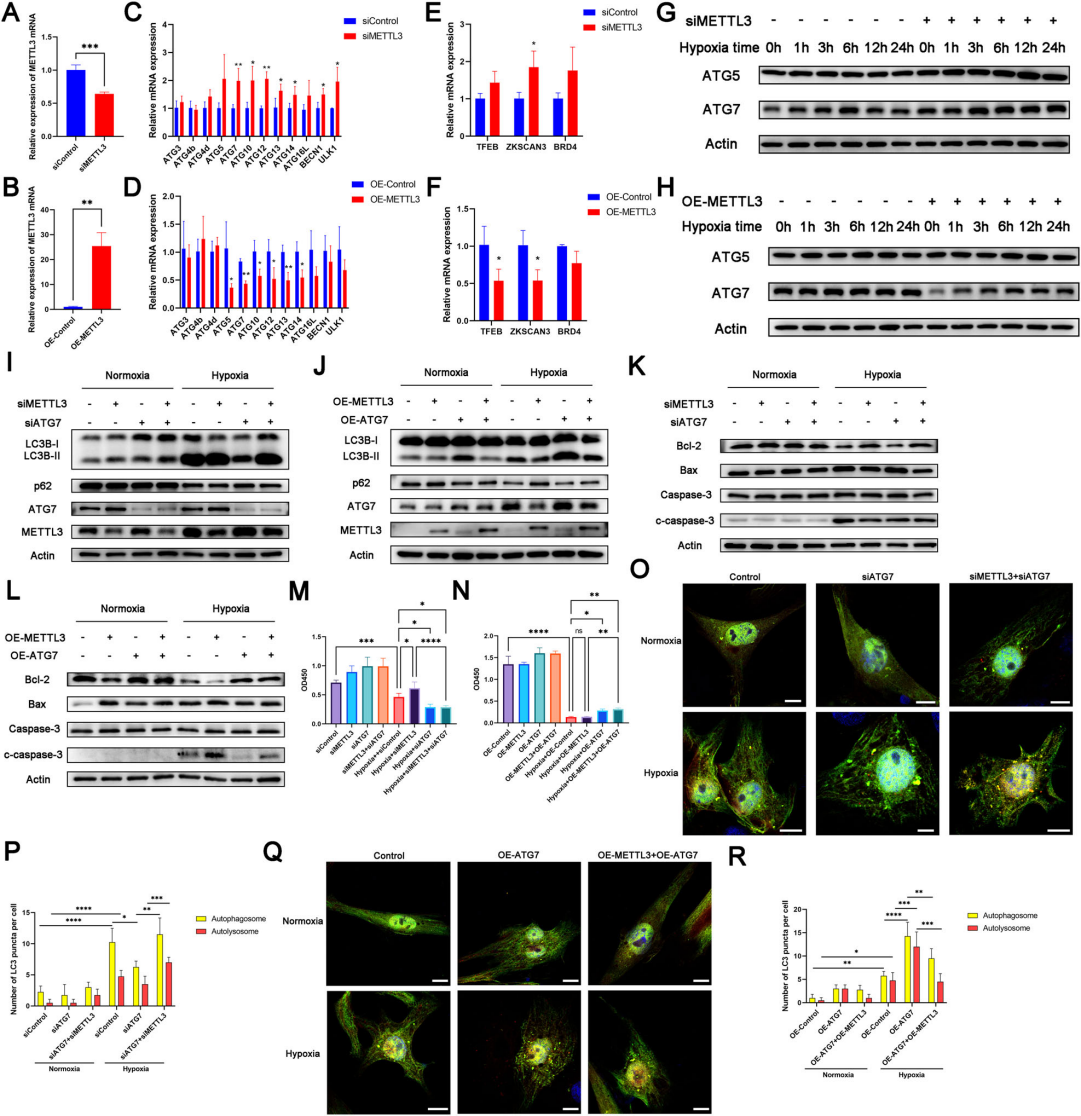
METTL3 affected autophagy through targeting ATG7. **A**, **C**, **E** mRNA levels of METTL3, ATGs (autophagy-related genes), and autophagy-related transcription factors in control and METTL3 knockdown cells under hypoxic conditions (*n* = 3). **B**, **D**, **F** mRNA levels of METTL3, ATGs (autophagy-related genes), and autophagy-related transcription factors in control and METTL3 overexpressing cells under hypoxic conditions (*n* = 3). **G** Western blot analysis of protein levels in H9c2 cells with METTL3 knockdown following hypoxia (*n* = 3). **H** Western blot analysis of protein levels in H9c2 cells with METTL3 overexpression following hypoxia (*n* = 3). **I**, **K** Western blot analysis of protein levels in H9c2 cells after transfection with or without METTL3 siRNA followed by treatment with or without ATG7 siRNA under the normoxic or hypoxic conditions (*n* = 3). **M** Cell viability was evaluated with the CCK8 assay (*n* = 5). **O**, **P** Immunofluorescence images of LC3 puncta in NRCMs treated in the same method as described above (*n* = 5). Scale bar: 10 μm. **J**, **L** Western blot analysis of protein levels in H9c2 cells after transfection with or without Ad-METTL3-OE followed by treatment with or without ATG7 overexpression plasmids under the normoxic or hypoxic conditions (*n* = 3). **N** Cell viability was evaluated with the CCK8 assay (*n* = 5). **Q**, **R** Immunofluorescence images of LC3 puncta in NRCMs treated in the same method as described above. Scale bar: 10 μm. All data were presented as mean ± SD. ns represents *p* > 0.05, ^*^p < 0.05, ^**^p < 0.01, ^***^p < 0.001, ^****^p < 0.0001.

To identify potential target genes of autophagy regulated by METTL3, we first used RT-qPCR to analyze the mRNA expression of autophagy-related genes in ischemic and hypoxic H9c2 cells. The results showed that METTL3 knockdown significantly increased the mRNA level of ATG7, while METTL3 overexpression decreased its mRNA level (Fig. 3A–D). Consistently, both METTL3 knockdown and overexpression significantly altered ATG7 protein levels in H9c2 cells (Fig. 3G–H) and NRCMs (Fig. S3A, B), respectively. Similar trends were observed in AC16 cells following METTL3 knockdown (Fig. S3C). However, some other autophagy-related proteins, such as ULK1, ATG5, and ATG12, did not show significant changes. Additionally, there were no significant changes in several important transcription factors associated with autophagy (Fig. 3E, F). Moreover, we were also surprised to find that although METTL3 was significantly elevated in hypoxic cardiomyocytes, the expression of ATG7 protein in the hypoxic group did not decrease due to the increase in METTL3 when compared to the normoxic group. In fact, many studies have reported that overall autophagy in cardiomyocytes increases significantly after hypoxia, which includes the key autophagy protein ATG7, and this mechanism involves multiple molecules regulating autophagy. As one of the molecules involved in the regulation of autophagy in cardiomyocytes after hypoxia, our study showed that METTL3 affected the expression of autophagy and its related factor ATG7 to some extent. Thus, despite the increased expression of METTL3 in cardiomyocytes after hypoxia, their overall autophagy levels, including the expression of ATG7, remained elevated. This suggested that the increase in METTL3 alone was not sufficient to fully reverse this process, which also explained the phenomenon where METTL3 and autophagy both increased in cardiomyocytes during acute ischemia and hypoxia.

To explore the clear relationship between METTL3 and ATG7 in autophagy, we treated H9c2 cells with ATG7 siRNA, and confirmed the knockdown efficiency by Western blotting. The results showed that the ratio of LC3B-II/LC3B-I decreased and p62 increased after ATG7 knockdown, while the protein expression of METTL3 did not change (Fig. 3I). In addition, ATG7 knockdown significantly inhibited the activity of cardiomyocytes (Fig. 3M), indicating the crucial role of ATG7 in autophagy regulation in H9c2 cells.

To confirm whether METTL3 affected autophagy by modulating the expression of ATG7, we conducted rescue experiments. We observed that silencing ATG7 reversed the upregulation of LC3B-II/LC3B-I and downregulation of p62 in METTL3 knockdown H9c2 cells, while overexpressing ATG7 reversed the downregulation of LC3B-II/LC3B-I and upregulation of p62 in METTL3-overexpressing H9c2 cells (Fig. 3I, J). Immunofluorescence analysis yielded consistent results (Fig. 3O–R). In addition, we also found METTL3 knockdown significantly inhibited the injury of ischemic and hypoxic cardiomyocytes and this effect was reversed by introducing ATG7 siRNA. Similarly, ATG7 overexpression in cardiomyocytes significantly reversed the injury induced by METTL3 overexpression in ischemic and hypoxic cardiomyocytes (Fig. 3K–N). In summary, these findings supported that METTL3 regulated autophagy by mediating ATG7.

To investigate whether this phenomenon occurs in other inducible autophagy, we attempted to verify the effects of METTL3 overexpression on autophagy in H9c2 cells under starvation induction and rapamycin treatment, respectively. We found that METTL3 overexpression inhibited autophagy induced by both starvation and rapamycin treatment. However, after ATG7 overexpression, the rescue of autophagy inhibition did not occur in rapamycin-treated cells, while autophagy was partially restored in starvation-treated cells (Fig. S3D–G). In conclusion, we still believe that the effect of METTL3 on autophagy and its underlying mechanisms may vary in different cells and under different stress conditions.

### METTL3 regulated ATG7 mRNA expression in an m^6^A-dependent manner

**Fig. 4:**
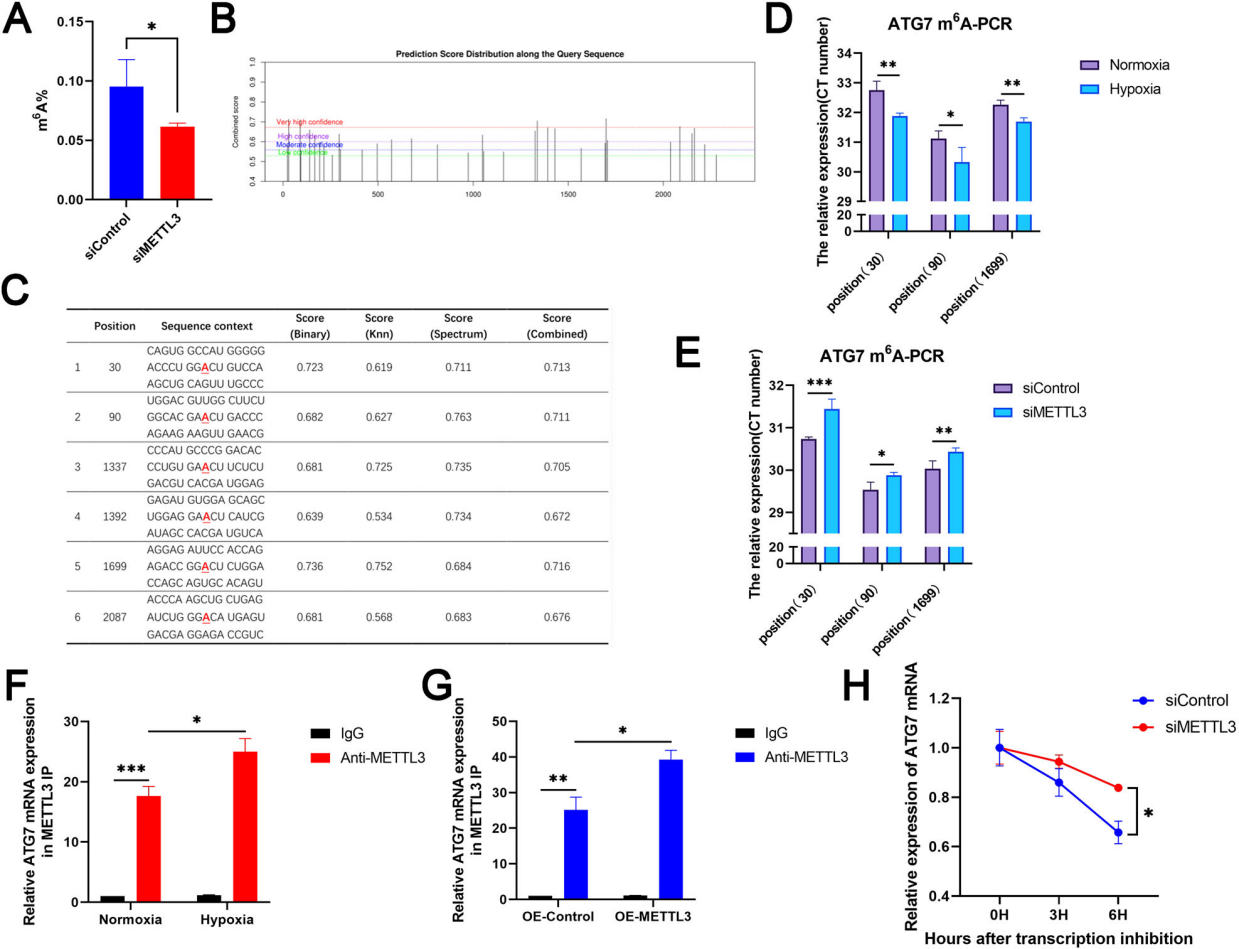
METTL3 regulated ATG7 expression in an m^6^A-dependent manner. **A** LC-MS/MS quantification of the m^6^A/A in mRNA of control and METTL3 knockdown cells under hypoxic conditions (*n* = 3). **B**, **C** The predicted m^6^A sites of ATG7 mRNA in rats by SRAMP. The red base sequence represents the m^6^A modified sequence (RRACH), where the underlined “A” represents the methylated adenine. **D** SMRAP was used to predict the m^6^A sites of ATG7 mRNA in H9c2 cells and SELECT technology was used to examine the levels of m^6^A in H9c2 cells with or without hypoxia (*n* = 3). **E** SELECT technology was used to examine the levels of m^6^A in control and METTL3 knockdown cells under hypoxic conditions (*n* = 3). **F** RNA immunoprecipitation-qPCR (RIP-qPCR) analysis of the interaction of ATG7 with METTL3 in H9c2 cells with or without hypoxia. Enrichment of ATG7 was measured by qPCR and normalized to input (*n* = 3). **G** RNA immunoprecipitation-qPCR (RIP-qPCR) analysis of the interaction of ATG7 with METTL3 in H9c2 cells transfected with or without Ad-METTL3-OE. Enrichment of ATG7 was measured by qPCR and normalized to input (*n* = 3). **H** H9c2 cells transfected with or without METTL3 siRNA were treated with 5 μg/ml actinomycin D for the indicated times (*n* = 3). All data were presented as mean ± SD. ns represents *p* > 0.05, ^*^p < 0.05, ^**^p < 0.01, ^***^p < 0.001, ^****^p < 0.0001.

To investigate whether METTL3 mediated m^6^A modification in ischemic and hypoxic cardiomyocytes, we used LC-MS/MS technique to detect the level of m^6^A modification in METTL3 knockdown H9c2 cells under ischemic and hypoxic conditions (Fig. 4A). We observed a decrease in the m^6^A modification level in METTL3 knockdown H9c2 cells, suggesting that METTL3 mediated m^6^A modification in cardiomyocytes induced by ischemia and hypoxia.

To explore whether METTL3 affected autophagy through m^6^A methylation modification of ATG7 mRNA, we first verified the presence of METTL3 binding sites for ATG7 mRNA. Through bioinformatics analysis, we predicted the most likely m^6^A modification sites to be bound (Fig. 4B, C). Secondly, using the SELECT technique that compared with the normoxic group, we confirmed that hypoxia significantly increased the content of ATG7 mRNA after m^6^A modification at the corresponding site (Fig. 4D). Additionally, METTL3 knockdown significantly decreased the content of ATG7 mRNA after m^6^A modification at the corresponding site (Fig. 4E). RNA immunoprecipitation and gene-specific qPCR (RIP-qPCR) experiments demonstrated the interaction between METTL3 and ATG7 mRNA in H9c2 cells, indicating that ATG7 mRNA was a direct target of METTL3, and both hypoxia and METTL3 overexpression increased the binding of ATG7 transcripts with METTL3 (Fig. 4F, G). In addition, mRNA stability analysis showed that the deletion of METTL3 prolonged the decline and stability of ATG7 mRNA transcripts in H9c2 cells (Fig. 4H). These results suggested that METTL3 targeted ATG7 transcription and regulated its expression in an m^6^A-dependent manner, thereby further regulating autophagy.

### YTHDF2 mediated mRNA expression of ATG7 through m^6^A-dependent mechanism

**Fig. 5:**
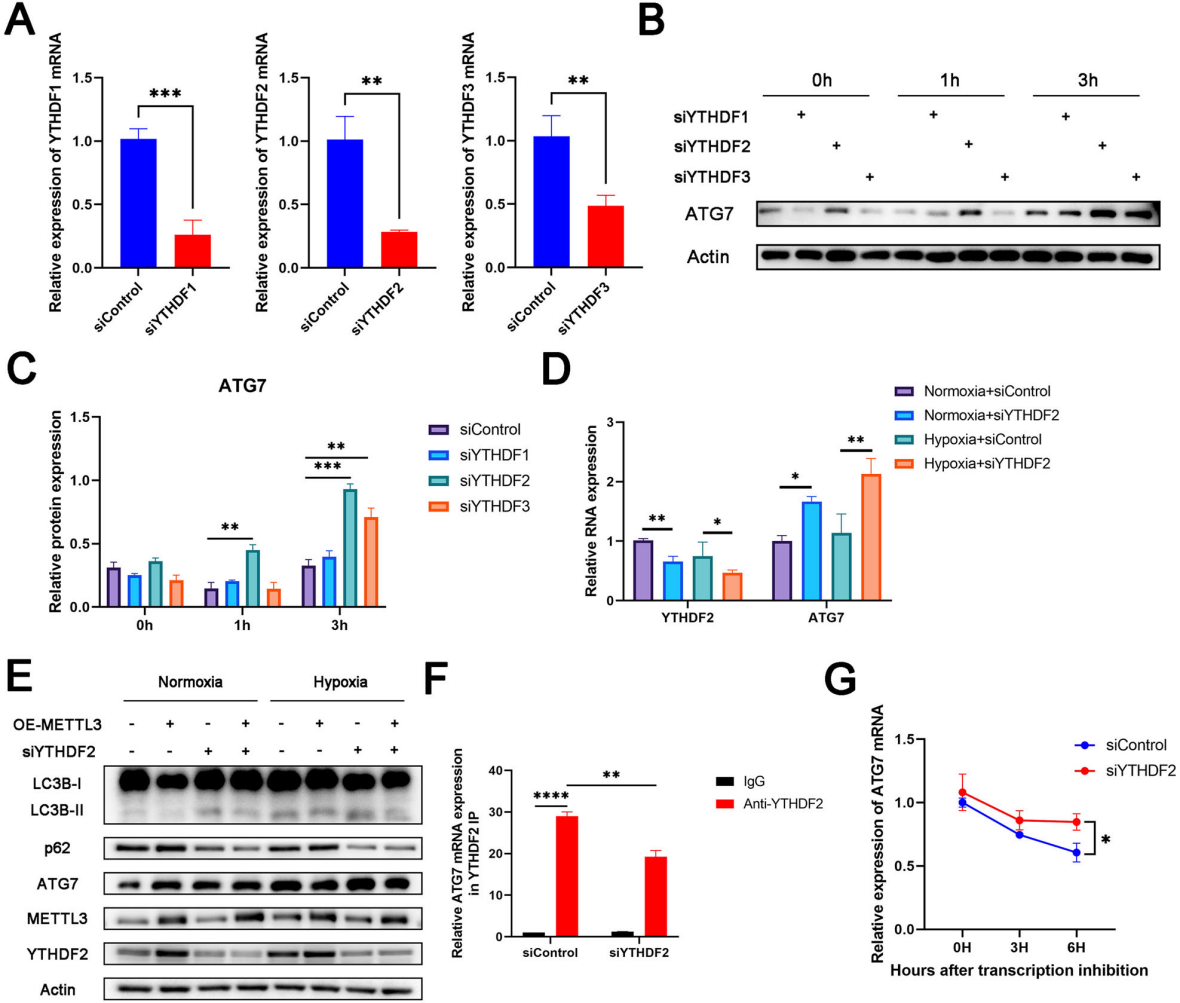
YTHDF2 mediated mRNA expression of ATG7 through m^6^A-dependent mechanism. **A** mRNA levels of YTHDF1, YTHDF2, and YTHDF3 in control and YTHDF1 knockdown or YTHDF2 knockdown or YTHDF3 knockdown cells (*n* = 3). **B**, **C** Western blot analysis of protein levels in control and YTHDF1 knockdown or YTHDF2 knockdown or YTHDF3 knockdown cells for the indicated times (*n* = 3). **D** mRNA levels of YTHDF2 and ATG7 in control and YTHDF2 knockdown cells with or without hypoxia (*n* = 3). **E** Western blot analysis of protein levels in H9c2 cells after transfection with or without Ad-METTL3-OE followed by treatment with or without YTHDF2 siRNA under the normoxic or hypoxic conditions (*n* = 3). **F** RNA immunoprecipitation-qPCR (RIP-qPCR) analysis of the interaction of ATG7 with METTL3 in H9c2 cells transfected with or without YTHDF2 siRNA. Enrichment of ATG7 was measured by qPCR and normalized to input (*n* = 3). **G** H9c2 cells transfected with or without YTHDF2 siRNA were treated with 5 μg/ml actinomycin D for the indicated times (*n* = 3). All data were presented as mean ± SD. ns represents *p* > 0.05, ^*^p < 0.05, ^**^p < 0.01, ^***^p < 0.001, ^****^p < 0.0001.

Previous studies have shown that a class of methylated reading proteins in m^6^A RNA methylation modification can affect the stability, splicing, nuclear transport, translation, and degradation of RNA [21]. Among them, YT521-B homologous (YTH) domain family proteins (YTHDF1-3) are the most studied reading proteins. It has been reported that YTHDF2 can selectively recognize and degrade m^6^A-modified mRNA, while YTHDF1 can promote the translation of mRNA [22, 23]. In addition, YTHDF3 mediates its interaction with YTHDF1 or YTHDF2 [24]. To identify the primary YTHDF protein involved in mediating ATG7 transcripts, we used small interfering RNA to knock down the mRNA expression of YTHDF1, YTHDF2, and YTHDF3, respectively. (Fig. 5A). Compared to the control group, only the YTHDF2 knockdown group showed significantly higher ATG7 protein levels at each hypoxia time point (Fig. 5B, C). Similarly, RT-qPCR also confirmed this result at the transcriptional level (Fig. 5D). Based on these results, we speculated that METTL3-mediated m^6^A modification of ATG7 mRNA might be selectively recognized by YTHDF2 and promote its degradation. To investigate this hypothesis, we conducted a rescue experiment and found that YTHDF2 knockdown reversed the downregulation of ATG7 and LC3B-II/LC3B-I ratio and upregulation of p62 in METTL3-overexpressing H9c2 cells (Fig. 5E), indicating that METTL3 regulated the mRNA expression of ATG7 in an m^6^A-dependent and YTHDF2-mediated manner. Furthermore, we further confirmed the interaction between ATG7 mRNA and YTHDF2 in hypoxia-treated H9c2 cells by RIP-qPCR experiment (Fig. 5F).

Subsequently, mRNA stability analysis showed that the deletion of YTHDF2 prolonged the decline and stability of ATG7 mRNA transcripts in H9c2 cells (Fig. 5G). To sum up, YTHDF2 regulated the mRNA expression of ATG7 through an m^6^A-dependent mechanism.

### METTL3 regulated MI-induced heart injury through ATG7-dependent autophagy in vivo

**Fig. 6:**
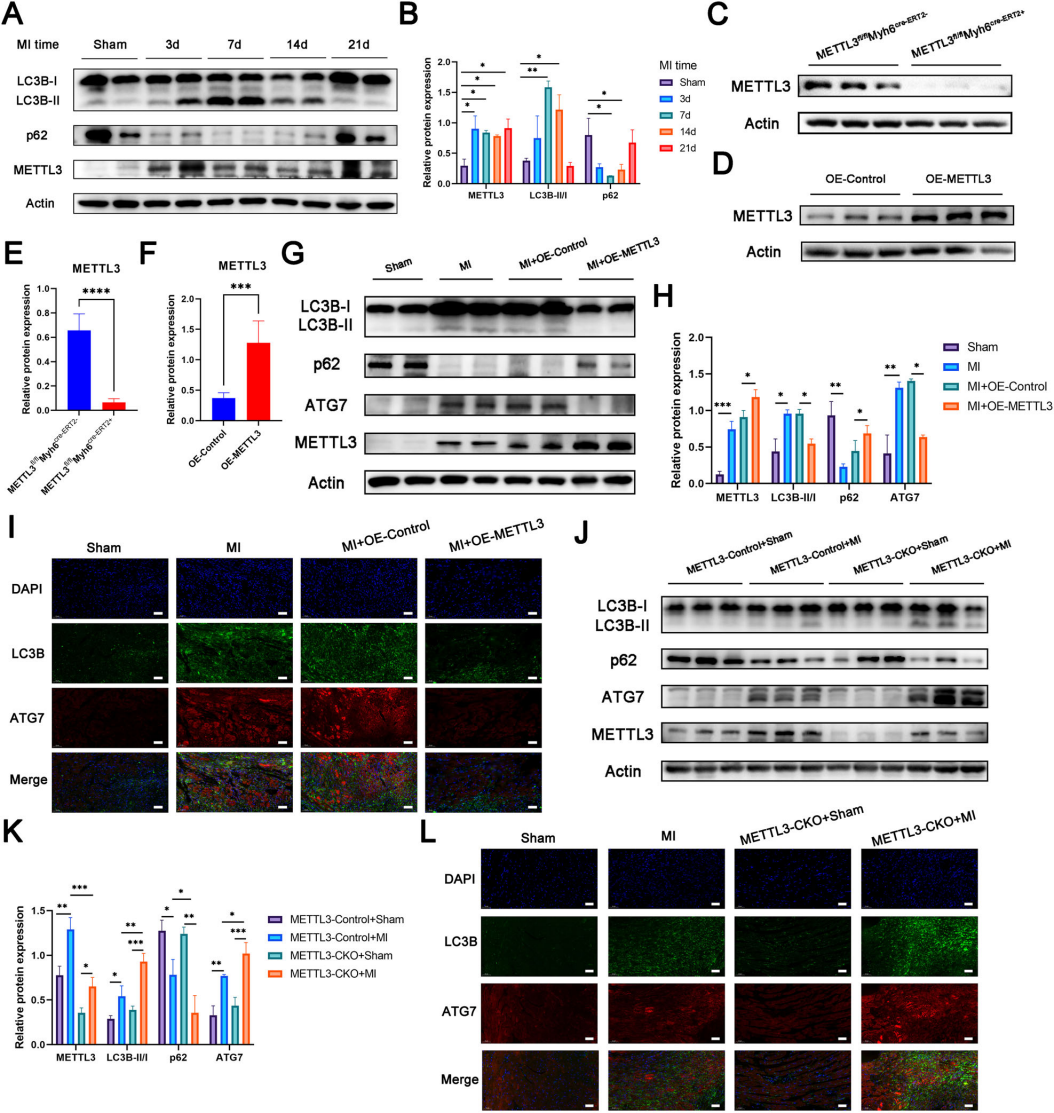
METTL3 regulated MI-induced heart injury through ATG7-dependent autophagy in vivo. **A**, **B** Western blot analysis of protein levels in the hearts of control mice (sham) or post-MI mice at 3 days, 1, 2, and 3 weeks after MI surgery (*n* = 5). **C**, **E** Western blot analysis of protein levels in hearts from METTL3flox/flox (METTL3-Control) or induced cardiac-specific METTL3 knockout (METTL3-CKO) mice (*n* = 5). **D**, **F** Western blot analysis of protein levels in hearts from mice after cardiac in situ injection with AAV9-NC (OE-Control) or AAV9-METTL3 (OE-METTL3) (*n* = 5). **G**, **H** Western blot analysis of protein levels in hearts isolated from OE-Control or OE-METTL3 mice at 1 week after MI surgery (*n* = 5). **I** The representative images of double fluorescent immunostaining for LC3B and ATG7 in hearts isolated from OE-Control or OE-METTL3 mice at 1 week after MI surgery (*n* = 5). Scale bar: 50 μm. **J**, **K** Western blot analysis of protein levels in hearts isolated from METTL3-Control or METTL3-CKO mice at 1 week after MI surgery (*n* = 5). **L** The representative images of double fluorescent immunostaining for LC3B and ATG7 in hearts isolated from METTL3-Control or METTL3-CKO mice at 1 week after MI surgery (*n* = 5). Scale bar: 50 μm. All data were presented as mean ± SD. ns represents *p* > 0.05, ^*^p < 0.05, ^**^p < 0.01, ^***^p < 0.001, ^****^p < 0.0001.

It has been reported that after permanent coronary artery ligation in mice, the autophagy signal is strongest in the first week and then begins to decline [25]. Our experimental results also confirmed this trend (Fig. 6A, B). To investigate the impact of METTL3 on autophagy in vivo, we constructed conditionally induced cardiac-specific METTL3 knockout mice (METTL3-CKO) (Fig. 6C, E); meanwhile, we constructed a mouse model with METTL3 overexpression by in situ injection of AAV9 adeno-associated virus into the heart (Fig. 6D, F). Following induction of myocardial infarction, we observed decreased protein expression of LC3B-II/LC3B-I and ATG7, alongside increased expression of p62, in cardiac tissues of METTL3-overexpressing mice compared to controls, indicating suppressed autophagy levels (Fig. 6G, H). In contrast, METTL3-CKO mice showed the opposite trend (Fig. 6J, K). Immunofluorescence staining of heart tissue sections provided consistent findings (Fig. 6I, L).

**Fig. 7:**
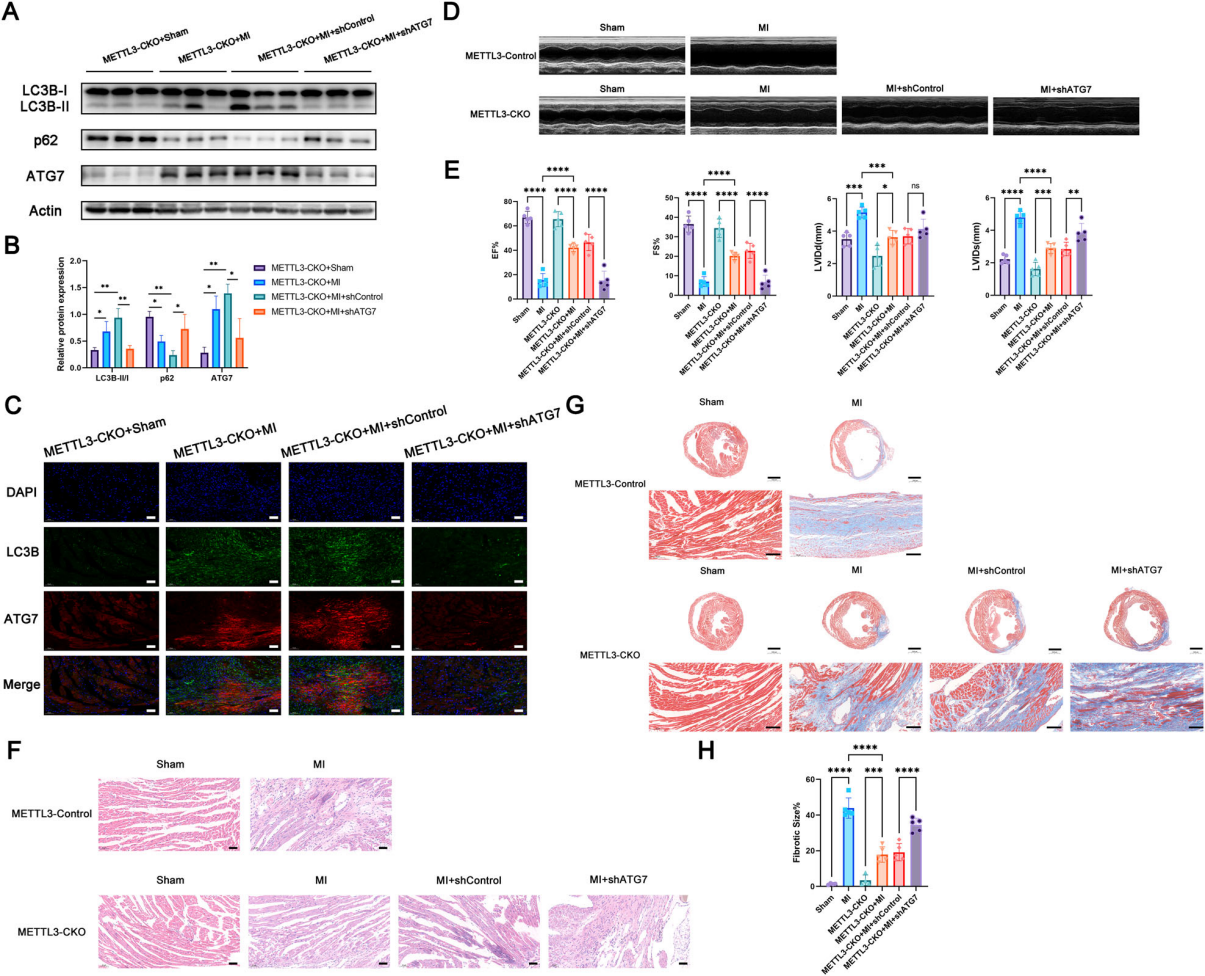
METTL3 regulated MI-induced heart injury through ATG7-dependent autophagy in vivo. **A**, **B** Western blot analysis of protein levels in hearts isolated from METTL3-CKO mice after cardiac in situ injection with AAV9-shControl or AAV9-shATG7 at 1 week after MI surgery (*n* = 5). **C** The representative images of double fluorescent immunostaining for LC3B and ATG7 in hearts isolated from METTL3-CKO mice after cardiac in situ injection with AAV9-shControl or AAV9-shATG7 at 1 week after MI surgery (*n* = 5). Scale bar: 50 μm. **D** Representative photographs of M-mode echocardiography 1 week post-MI (*n* = 5). **E** Quantitative analysis of echocardiography. FS fractional shortening, EF ejection fraction, LVIDd left ventricular internal diameter end-diastolic, LVIDs left ventricular internal diameter end-systolic (*n* = 5). **F** Hematoxylin and eosin (HE) staining of heart tissue sections 3 weeks post-MI (*n* = 5). Scale bar: 50 μm. **G** Masson Trichrome staining of heart tissue sections 3 weeks post-MI (*n* = 5). Scale bar: 1000 μm or 100 μm. **H** Percentage of left ventricle area occupied by scar tissue (*n* = 5). All data were presented as mean ± SD. ns represents *p* > 0.05, ^*^p < 0.05, ^**^p < 0.01, ^***^p < 0.001, ^****^p < 0.0001.

Furthermore, we constructed a mouse model with ATG7 knockdown in METTL3-CKO mice via in situ injection of AAV9 adeno-associated virus into the heart for rescue experiments. Our results demonstrated that ATG7 knockdown reversed the elevated autophagy levels observed in the hearts of METTL3-CKO mice following myocardial infarction (Fig. 7A, B). Immunofluorescence staining of heart tissue sections provided additional support for these findings (Fig. 7C). It was suggested that METTL3 interfered with the expression of ATG7, thus affecting the autophagy of cardiomyocytes.

To further elucidate the impact of METTL3 on myocardial infarction severity and cardiac function in mice, we assessed cardiac function using echocardiography one week after permanent coronary artery ligation. Compared with the control group, METTL3-CKO mice exhibited a significant improvement in cardiac function, which was reversed upon ATG7 knockdown (Fig. 7D, E). Subsequent histological analyses via HE staining and Masson trichrome staining revealed that METTL3-CKO mice showed greater survival of cardiomyocytes and reduced myocardial fibrosis compared to controls, with these effects reversed upon ATG7 knockdown (Fig. 7F–H). These results suggested that METTL3 knockdown mitigated myocardial infarction severity and enhanced cardiac function in mice, at least in part by promoting ATG7 expression and thus autophagy.

To demonstrate the physiological importance of ATG7 expression, we injected AAV9-OE-Control and AAV9-OE-ATG7 into the hearts of mice, respectively, and performed coronary artery ligation on the mice three weeks later. We found that in mice with myocardial infarction, overexpression of ATG7 could improve cardiac function to some extent (Fig. S4A, B), although the reduction in myocardial fibrosis area was slight and not statistically significant (Fig. S4C–E). These findings suggested that overexpression of ATG7 could partially protect cardiac function in mice with myocardial infarction.

## Discussion

**Fig. 8:**
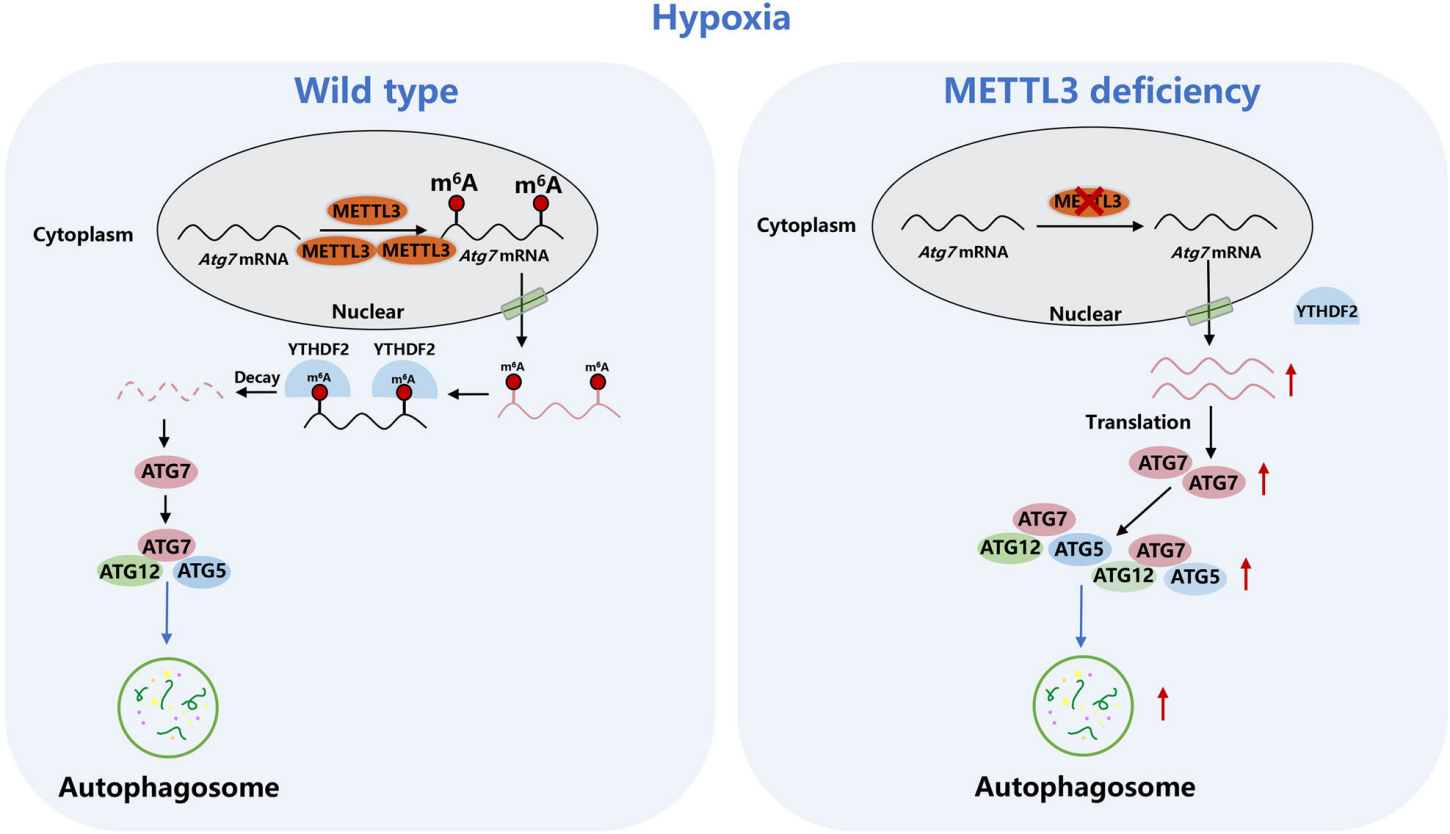
Schematic representation of mechanisms by which METTL3 regulates autophagy of hypoxia-induced cardiomyocytes. Methyltransferase METTL3 regulates autophagy through m^6^A methylation of autophagy-related factor ATG7, which affects the survival of hypoxic cardiomyocytes.

The present study uncovered that METTL3 negatively regulated the autophagy of cardiomyocytes treated with ischemia and hypoxia, thereby affecting the survival of cardiomyocytes. In addition, we found that ATG7 was a key target gene for METTL3 regulation. Under hypoxic conditions, METTL3 deficiency led to a decrease in the m^6^A modification level of ATG7 mRNA. After ATG7 mRNA exited the nucleus, its recognition and binding by YTHDF2 were reduced, allowing it to be further translated into protein to participate in autophagy. This enhanced the autophagy levels in ischemic and hypoxic cardiomyocytes, ultimately promoting cell survival (Fig. 8). Based on the above experiments, we discussed the regulation of autophagy-related factor ATG7 by METTL3 m^6^A methylation modification, and proposed the important role of METTL3 in ischemic and hypoxic cardiomyocytes.

Autophagy is a type II programmed cell death. It has many manifestations, but all follow the autophagy—lysosome pathway in which cytoplasmic substances are transported to lysosomes for degradation. Autophagy flux is a dynamic process in which these steps occur continuously in the cell. If any of these links is disrupted, autophagy will not be able to complete its biological function [26]. The activation of autophagy flux may be caused by either the increase of autophagosomes or the failure of autolysosome clearance. In our study, we used Bafilomycin-A1 to treat cells and found that METTL3 knockdown activated autophagy mainly by promoting autophagosome synthesis. While baseline autophagy exerts a protective and mitigating effect in ischemic injury, severe ischemia, and hypoxia conditions can lead to excessive induction of cardiac autophagy, potentially exacerbating cardiomyocyte death and deteriorating cardiac function [27]. Interestingly, compared with other forms of cell injury, autophagy and apoptosis seem to be more closely related, and the pathway and mechanism between them have been more thoroughly studied. They can influence each other and become an important regulator of coronary heart disease [28]. A previous study has shown that co-culture with bone marrow mesenchymal stem cells up-regulated ALKBH5 to protect FIP200 mRNA from YTHDF2-mediated degradation, thus promoting autophagy and inhibiting apoptosis of compressed neural stem cells [29]. In addition, another study found that HIF-1α-overexpressed extracellular vesicles of mesenchymal stem cells-derived activated YTHDF1-mediated protective autophagy, inhibiting apoptosis and senescence of pancreatic β cells under hypoxia [30]. In our study, 3-MA treatment enhanced cell damage mediated by the overexpression of METTL3, while Rapamycin reduced myocardial injury induced by ischemia and hypoxia in cardiomyocytes with overexpression of METTL3. Inhibiting the expression of METTL3 may be one strategy to inhibit ischemic and hypoxic myocardial injury in the future.

In the process of autophagy, ATG7 activates the ATG12 protein and participates with ATG10 to promote the covalent connection between ATG12 and ATG5. Subsequently, ATG12-ATG5 reacts with ATG16L to form homo-oligomeric [31], which is required for autophagosome formation [32]. Our results suggested that METTL3 regulated ATG7 mRNA expression in an m^6^A-dependent manner, thus affecting autophagy. A similar phenomenon was found in a recent study where METTL3 regulated autophagy by affecting the stability of ATG7 transcripts in a m^6^A-dependent manner, thereby affecting synovial cell senescence in osteoarthritis [33]. However, another study found that in hepatoma cells resistant to Sorafenib, the downregulation of METTL3 led to the degradation of FOXO3 and the upregulation of autophagy proteins such as ATG5, ATG7, and ATG16L1, promoting autophagy-induced drug resistance of hepatocellular carcinoma cells to Sorafenib [34]. This difference may be due to the specificity of m^6^A methylation in autophagy in different types of tissues or cells.

METTL3 is a methyltransferase that catalyzes m^6^A RNA methylation modification [35, 36]. In hypoxia-reoxygenated cardiomyocytes, overexpression of METTL3 suppressed the expression of transcription factor TFEB, thus inhibiting autophagy and causing cardiomyocyte injury [17]. On the contrary, β-elemene induced METTL3 to positively regulate autophagy in non-small cell lung cancer resistant to Gefitinib [37]. Our study found that under the condition of ischemia and hypoxia, METTL3 knockdown promoted cardiomyocyte autophagy, while overexpression of METTL3 inhibited cardiomyocyte autophagy. In other words, autophagy mediated by METTL3 plays different roles in different tissues or cells. Of course, it may also behave differently under different environmental stimuli. mRNA transcripts with m^6^A modification have different endings due to the recognition of different m^6^A binding proteins [38, 39]. YTHDF2 and YTHDF3 cooperate to promote the degradation of target mRNAs, thus reducing the expression of target genes; YTHDF1 and YTHDF3 cooperate to promote the translation of target mRNAs, thus promoting the expression of target genes [40, 41]; IGF2BPs can enhance the stability of mRNAs, thus improving translation efficiency [42]. Our results suggested that YTHDF2 mediated the stability of ATG7 mRNA in a m^6^A-dependent manner and ATG7 mRNA was the target gene of YTHDF2.

In recent years, there has been increasing interest in investigating the relationship between METTL3-mediated m^6^A RNA methylation and ischemic heart diseases. However, the effects of METTL3 on these diseases appear to vary significantly under different conditions. For instance, METTL3 affected the processing and maturation of let-7e and miR-17-92 clusters to promote the formation of restorative neovascularization around myocardial infarction, thus improving cardiac function, suggesting that METTL3 was a favorable factor in myocardial infarction [43]. Conversely, another study observed that overexpression of METTL3 reduced cardiomyocyte apoptosis induced by H/R and cardiac injury induced by I/R, suggesting that METTL3 was one of the factors leading to ischemia-reperfusion injury [17]. In this study, we found that METTL3 had a detrimental effect in the context of myocardial infarction. In fact, METTL3-mediated m^6^A RNA methylation may be a double-edged sword for ischemic heart diseases. Some genes promote the development of the disease after methylation, while others promote the development of the disease after demethylation [44]. In a word, m^6^A modification does participate in the pathophysiological process of ischemic heart diseases, and it is necessary to maintain the normal structure and function of the heart. However, the exact mechanism of m^6^A modification in ischemic heart diseases remains unclear and requires further exploration.

The current study has several limitations. Firstly, we mainly focused on ATG7, a key molecule affected by METTL3, while other autophagy-related factors associated with METTL3 need to be further explored. In addition, this study was limited to cell and animal experiments, and lacked the support of clinical samples, which is the point that needs to be further studied. Lastly, the mechanism of METTL3-mediated m^6^A methylation of ATG7 has not been fully clarified, and its performance may be different in different environments or cells, which is also a point to be studied in the future.

In conclusion, this study reveals the critical role of METTL3 in mediating the autophagy of ischemic and hypoxic cardiomyocytes. METTL3 affects the fate of ischemic and hypoxic cardiomyocytes by regulating autophagy and affecting the stability of ATG7 transcripts in an m^6^A-YTHDF2-dependent manner. These findings provide new insights into the potential molecular mechanisms by which m^6^A modification regulates autophagy in cardiomyocytes, as well as into the development of strategies for the prevention and treatment of acute myocardial infarction.

## Materials and methods

### Cell culture and treatments

H9c2 rat cardiomyocyte cell line and AC16 human cardiomyocyte cell line were purchased from the Cell Bank of Type Culture Collection of the Chinese Academy of Sciences and were free of mycoplasma contamination. H9c2 and AC16 cells were cultured in high glucose DMEM (Gibco Life Technologies, USA) combined with FBS (10%, Clark Bioscience, USA), penicillin (100 U/ml), and streptomycin (100 µg/ml) in humidified air with 5% CO_2_ and 95% air at 37 °C. Neonatal rat cardiomyocytes (NRCMs) were obtained from the hearts of SD rats born within 1-2 days. Primary cultures of NRCMs were prepared as described previously [45]. NRCMs were cultured in DMEM-F12 combined with FBS (10%), penicillin (100 U/ml), and streptomycin (100 μg/ml) in a humidified atmosphere with 5% CO_2_ and 95% air at 37 °C. To establish the ischemia and hypoxia model, we replaced the culture medium with serum-free and glucose-free DMEM, and then placed the cells in a hypoxic incubator containing 95% N_2_ and 5% CO_2_. In addition, H9c2 cells were subjected to hypoxia for 3 h and NRCMs were subjected to hypoxia for 6 h.

### Cell transfection, plasmids, and RNA knockdown

Recombinant adenovirus-METTL3 (Ad-METTL3), recombinant adenovirus-mCherry-EGFP-LC3 (Ad-mCherry-EGFP-LC3), and the negative control adenovirus (Ad-NC) were purchased from Hanheng Biotechnology (Shanghai, China). Gene sequences coding for ATG7 were cloned into the pcDNA3.1 vector (Invitrogen). The METTL3 siRNA (siMETTL3), ATG7 siRNA (siATG7), YTHDF1 siRNA (siYTHDF1), YTHDF2 siRNA (siYTHDF2), YTHDF3 siRNA (siYTHDF3) and negative control siRNA (siNC) were also purchased from it. Cells in 6-well or 12-well culture plates were infected with adenovirus for 48 h. Similarly, cells were transfected with siRNA by use of Lipofectamine 3000 (Thermo Scientific, MA, USA) reagent for 48 h. The procedures were carried out strictly according to the manufacturer’s instructions. The METTL3 siRNA (rat) sequence was 5′-GGACUUAAGGAAUCCAGAATT-3′. The METTL3 siRNA (human) sequence was 5′-GCAAGAAUUCUGUGACUAUTT-3′. The ATG7 siRNA sequence was 5′-GGUCAAAGGACAAAGUUAATT-3′. The YTHDF1 siRNA sequence was 5′-GGACAUUGGUACUUGGGAUTT-3′. The YTHDF2 siRNA sequence was 5′-GGGAUUGACUUCUCAGCAUTT-3′. The YTHDF3 siRNA sequence was 5′-UAAGUCAAAGAAGACGUAUUAUU-3′ and NC sequence was 5′-UUCUCCGAACGUGUCACGUTT-3′.

### Animals

Newborn male SD rats and C57bl/6 mice (6-8 weeks) were purchased from Shanghai SLAC Laboratory Animals (Shanghai, China). The METTL3-iCKO mice constructed by CRISPR/Cas9 were obtained from the Shanghai Model Organisms Center (Shanghai, China). We detected the animal genotypes by analyzing tail genomic DNA. The mice were fostered under pathogen-free conditions. The animal care and experiments were approved by the Animal Care Ethics Committee of Zhongshan Hospital, Fudan University. In the animal experiments, each group consisted of 4–5 mice and the allocation of animals to each group was completely randomized. In addition, the investigators were informed about group assignments during the experiment and/or when assessing results.

### MI model

To construct a mouse with cardiac overexpression of METTL3 or ATG7, we exposed the heart of the mouse under intravenous anesthesia, and then injected 100 μl of AAV9-METTL3 or AAV9-ATG7 (Hanheng Biotechnology Co., Ltd. Shanghai, China) or relevant control into the mouse myocardial tissue through an insulin needle. Similarly, the mouse with cardiac ATG7 knockdown was also constructed like this, except that AAV9-ATG7 shRNA (Hanheng Biotechnology Co., Ltd. Shanghai, China) was injected in the METTL3-iCKO mouse. Three weeks later, they were anesthetized with 2.0% isoflurane (Baxter International Inc., Deerfield, IL, USA) and then subjected to endotracheal intubation to receive mechanical ventilation. Next, the heart was fully exposed and the left anterior descending artery ligated. The sham group was conducted to the same procedure but without ligation. One week after coronary artery ligation, we took the hearts of mice for protein extraction and immunofluorescence staining. Similarly, another group of mice were subjected to transthoracic echocardiography (Vevo 770 instrument, Visual Sonics Inc., Toronto, Canada) 3 weeks after coronary artery ligation. Then, animals were euthanized, and their hearts were removed and preserved in a 4% tissue fixative solution for subsequent staining. Paraffin-embedded sections were prepared and stained with hematoxylin-eosin (HE) and Masson trichrome.

### Western blot analysis

The total protein of cells and tissues was extracted by RIPA lysis buffer (Beyotime Biotechnology, P0013B) with protease and phosphatase inhibitor cocktail (Beyotime Biotechnology, P1045) on ice. Micro BCA™ Protein Assay Kits (Beyotime Biotechnology, P0012) were used to determine the protein concentration. About 30 μg total protein for each sample was loaded and separated by SDS-PAGE and then transferred to polyvinylidene difluoride membranes (Millipore, IPVH00010). The membranes were incubated with primary antibodies at 4 °C overnight after being blocked by 5% BSA at room temperature for 1 h. After washing three times, the membranes were incubated with goat anti-mouse or anti-rabbit HRP-conjugated secondary antibodies (Abcam, Cambridge, MA, USA 1:10,000) at room temperature for 1 h. The protein bands were visualized by a chemiluminescence imaging system. The primary antibodies used in this study are listed in Supplementary Table I.

### Quantitative real-time PCR (qPCR) analysis

Total RNA extracted from cells was performed with UNIQ-10 Column TRIzol Total RNA Isolation Kit (Sangon Biotech, Shanghai, China) and then reverse transcribed into cDNA using Hifair® II first Strand cDNA Synthesis Kit (gDNA digester plus; 11119ES60, Yeasen). qPCR analysis was conducted using Hifair® qPCR SYBR® Green Master Mix (No Rox; 11201ES08, Yeasen) on an Applied Biosystems 7500 Real-Time PCR System (Applied Biosystems, Foster City, CA, USA). The relative mRNA expression level was analyzed by the 2(-ΔΔCT) method and calculated using Actin as the normalization control. The sequences of primers used are presented in Supplementary Table II.

### Cell viability assay

A Cell Counting Kit-8 (CCK-8) assay (Beyotime Biotechnology, C0038) was performed to assess the cell viability of H9c2 cells. Cells were seeded in 96-well plates at a concentration of 5000 cells per well. After transfection or drug treatment, cells were incubated with 10 μl CCK-8 solution per well for 1.5 h at 37 °C. Finally, the 450 nm absorbance (OD) was measured by a microplate reader (Bio-Tek).

### Immunofluorescence analysis

Adenoviruses of EGFP-mCherry-LC3 (Hanheng Biotechnology Co., Ltd. Shanghai, China), a specific marker of autophagosome formation, were transfected into the cells cultured on coverslips. After 48 h of transfection, cells were fixed with 4% paraformaldehyde at room temperature for 10 min after treatment with hypoxia for 3 h. Finally, cells were observed under a confocal laser scanning microscope and images were collected after adding an anti-fluorescence attenuation sealer. The images of cells were analyzed by ImageJ software. Cells were detected by green (EGFP) or red (mCherry) fluorescence.

### Transmission electron microscopy (TEM)

Under the observation of transmission electron microscopy (TEM), autophagosome was characterized as a crescent-shaped or cup-shaped double or multilayered bubble-like structure with a tendency to enclose cytoplasmic components. Cells were digested, centrifuged and the supernatant discarded. After centrifugation, the cells were fixed for 30 min with 2.5% glutaraldehyde at room temperature, and then placed at 4 °C overnight. They were then incubated in 1% OsO_4_ in phosphate buffer (0.1 M, pH 7.0) for 2 h, dehydrated in graded ethanol, and saturated in graded acetone. The electron photomicrographs were taken from autophagosomes of cells under transmission electron microscopy (HITACHI HT7700).

### Quantification of mRNA m^6^A

Total RNA was extracted from H9c2 cells using TRIzol reagent (Sangon Biotech, Shanghai, China). Then, mRNA was purified using a Dynabeads mRNA DIRECT kit (Thermo Scientific, MA, USA) according to the manufacturer’s protocols. According to the concentration of mRNA, 50–100 ng mRNA was digested by nuclease P1 (1 U, Sigma) in 25 μl of buffer containing 20 mM of NH_4_OAc (pH = 5.3) at 42 °C for 2 h, followed by the addition of 3 μl NH_4_HCO_3_ (1 M) and alkaline phosphatase (1 U, Sigma) with incubation at 37 °C for 2 h. The sample was diluted to a total volume of 50 µl and filtered (0.22-μm pore size, 4-mm diameter, Millipore, SLGVR04NK). The overall m^6^A level of sample RNA was analyzed by LC-MS/MS. The ratio of m^6^A to A was calculated based on the measured concentrations.

### SELECT for detection of the m^6^A% in mRNA

We used a SELECT kit (Guangzhou EpiBiotek Co., Ltd., Guangzhou, China) to determine the m^6^A site of ATG7 mRNA bound by METTL3. We operated the detailed experimental scheme according to the instructions [46].

### RNA immunoprecipitation-qPCR (RIP-qPCR) analysis

According to the manufacturer’s instructions, RNA immunoprecipitation was undertaken with a RIP kit (GENESEED, China). The cells of different groups were first extracted and pretreated. Subsequently, each group was divided into three: IP group, IgG group, and Input group. The Input samples were stored at −80 °C. METTL3, YTHDF2, and IgG antibodies were conjugated to protein A/G magnetic beads in IP buffer overnight at 4 °C in a vertical mixer. The complex on the magnetic beads was eluted with the buffer in the kit. Finally, the precipitated RNA and the input RNA were extracted by the column method and then detected by RT-qPCR.

### mRNA stability analysis

In brief, cells were transfected with NC siRNA or YTHDF2 siRNA for 36 h and then treated with 4 μM actinomycin D (MedChemExpress, USA) to inhibit the global mRNA transcription. After incubation for the indicated points, the cells were collected and the total RNA was extracted for reverse transcription. The level of mRNA transcription was detected by RT-qPCR.

### Statistical analysis

The sample size for each group was set at three or more to ensure that pre-specified effect sizes could be effectively tested. All samples were randomly assigned to either the control group or the experimental group. The investigators were informed of the group assignment during the experiment and/or when assessing the results. Additionally, for each figure, the statistical tests were shown to be appropriate and the data met the assumptions of the tests. The variance of each group estimated by investigators was similar between the groups for which statistical comparisons were made. The data were represented as mean ± SD. Two-tailed unpaired Student’s *t*-test was used to compare two independent groups. One- or two-way ANOVA was used for multiple-group comparisons. Statistical analysis was performed with GraphPad Prism 9.0 (GraphPad, La Jolla, CA, USA). *p* values < 0.05 were considered significant statistically (^*^p < 0.05, ^**^p < 0.01, ^***^p < 0.001, ^****^p < 0.0001).

## Supplementary information

Supplementary Figures

Supplemental Material

Original Figures

## Data Availability

The data supporting the results of the study are available from the corresponding author upon reasonable request.
