# Supplementary material for: METTL3 regulates autophagy of hypoxia-induced cardiomyocytes by targeting ATG7
Source: Cell Death Discov. 2025 Feb 1;11:37. doi: 10.1038/s41420-025-02320-3 (PMC11787298; doi:10.1038/s41420-025-02320-3)
Supplement: Supplementary file 1 — Supplementary Figures [file 41420_2025_2320_MOESM1_ESM.pdf]

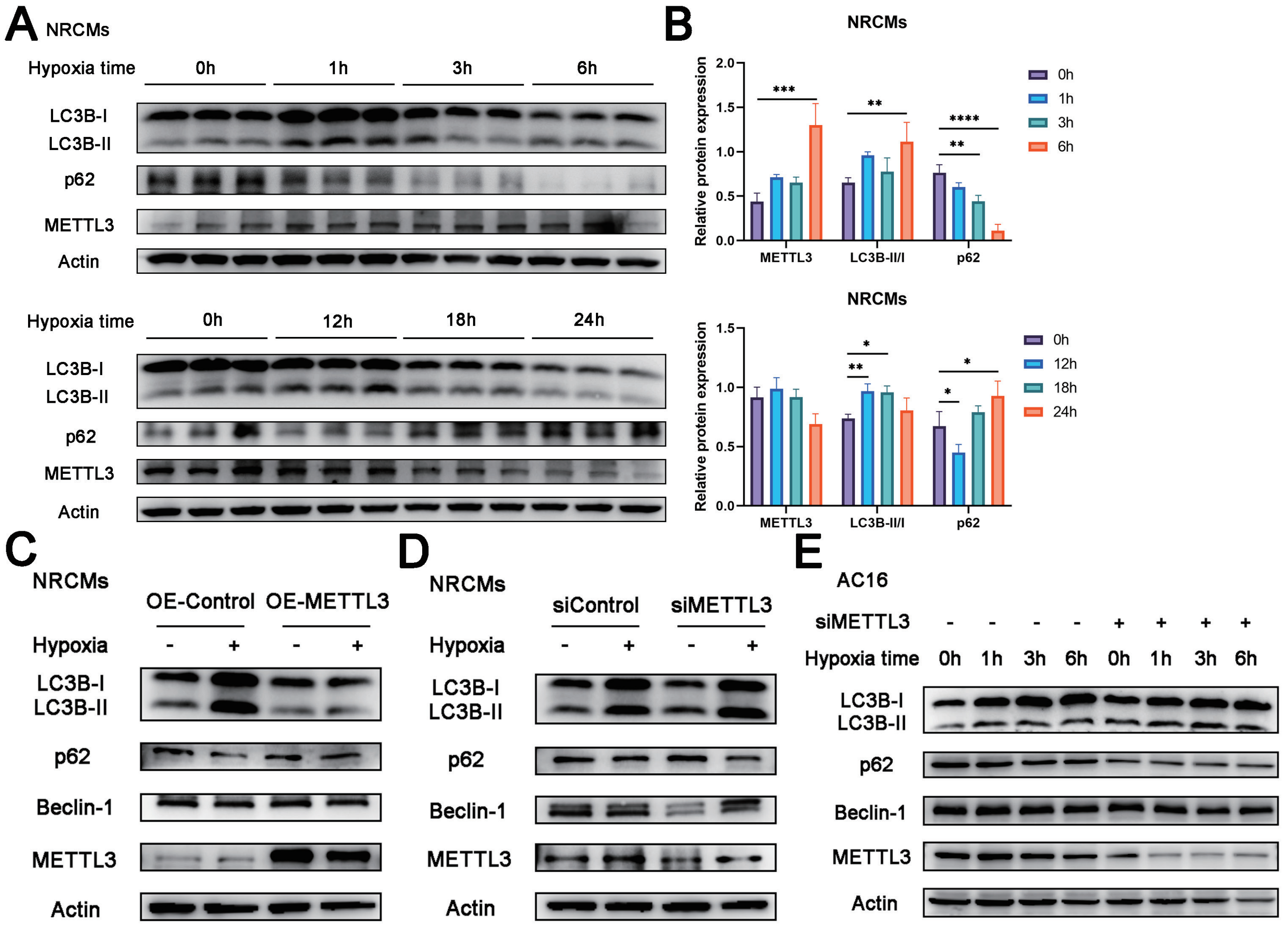

Figure S1. METTL3 regulated autophagy of hypoxia-induced cardiomyocytes.

(A, B) NRCMs were exposed to hypoxia for 0 h, 1 h, 3 h, 6 h, 12 h, 18 h and 24 h respectively, and the protein levels was analyzed by Western blotting (n=3). (C) Western blot analysis of protein levels in NRCMs with METTL3 overexpression following hypoxia for the indicated time periods (n=3). (D) Western blot analysis of protein levels in NRCMs with METTL3 knockdown following hypoxia for the indicated time periods (n=3). (E) Western blot analysis of protein levels in AC16 cells with METTL3 knockdown following hypoxia for the indicated time periods (n=3). All data were presented as mean±SD. ns represents $p > 0.05$, *$p < 0.05$, **$p < 0.01$, ***$p < 0.001$, ****$p < 0.0001$.

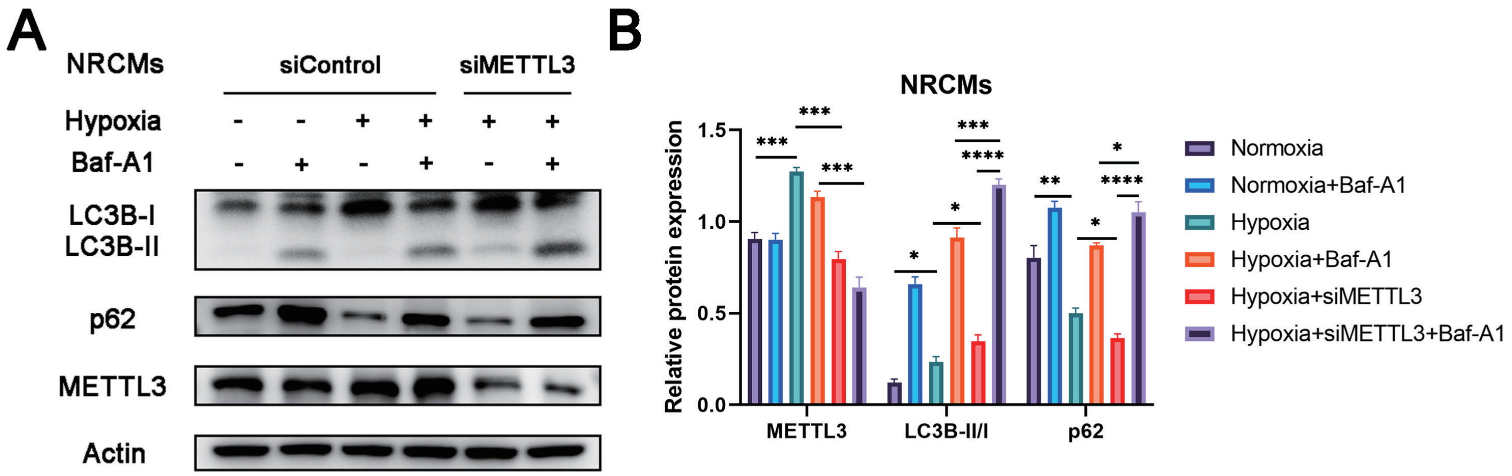

Figure S2. METTL3 affected the injury of hypoxic cardiomyocytes by autophagy.

(A, B) Western blot analysis of protein levels in control and METTL3 knockdown NRCMs. The cells were treated with or without Bafilomycin A1 (BafA1, 100 nM) for 3 h prior to incubation with or without hypoxia (n=3). All data were presented as mean±SD. ns represents p > 0.05, *p < 0.05, **p < 0.01, ***p < 0.001, ****p < 0.0001.

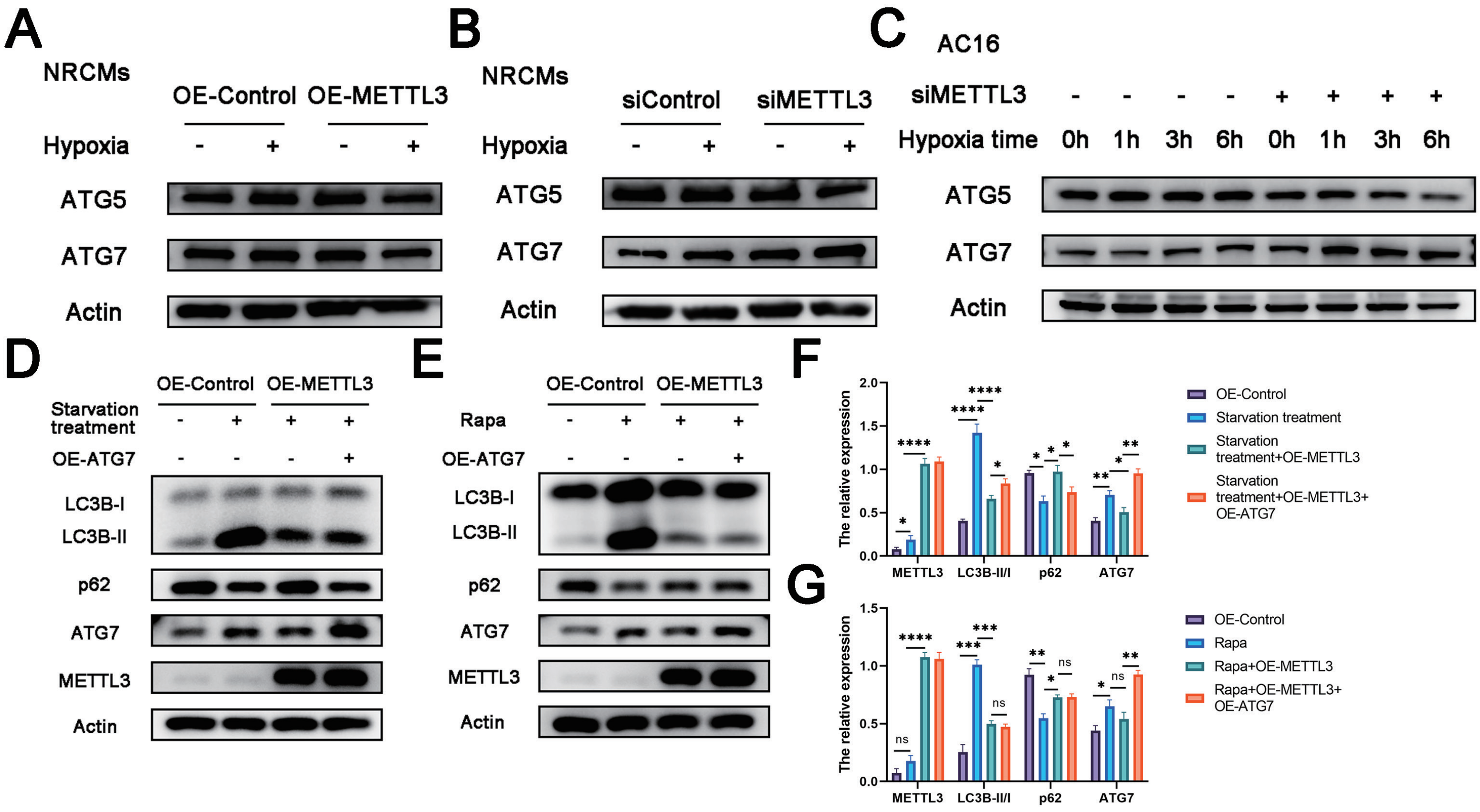

Figure S3. METTL3 affected autophagy through targeting ATG7.

(A) Western blot analysis of protein levels in NRCMs with METTL3 overexpression following hypoxia for the indicated time periods (n=3). (B) Western blot analysis of protein levels in NRCMs with METTL3 knockdown following hypoxia for the indicated time periods (n=3). (C) Western blot analysis of protein levels in AC16 cells with METTL3 knockdown following hypoxia for the indicated time periods (n=3). (D, F) Western blot analysis of protein levels in H9c2 cells after transfection with or without Ad-METTL3-OE followed by treatment with or without ATG7 overexpression plasmids under normal treatment or starvation treatment (n=3). (E, G) Western blot analysis of protein levels in H9c2 cells after transfection with or without Ad-METTL3-OE followed by treatment with or without ATG7 overexpression plasmids under normal treatment or rapamycin treatment (n=3). All data were presented as mean±SD. ns represents p > 0.05, *p < 0.05, **p < 0.01, ***p < 0.001, ****p < 0.0001.

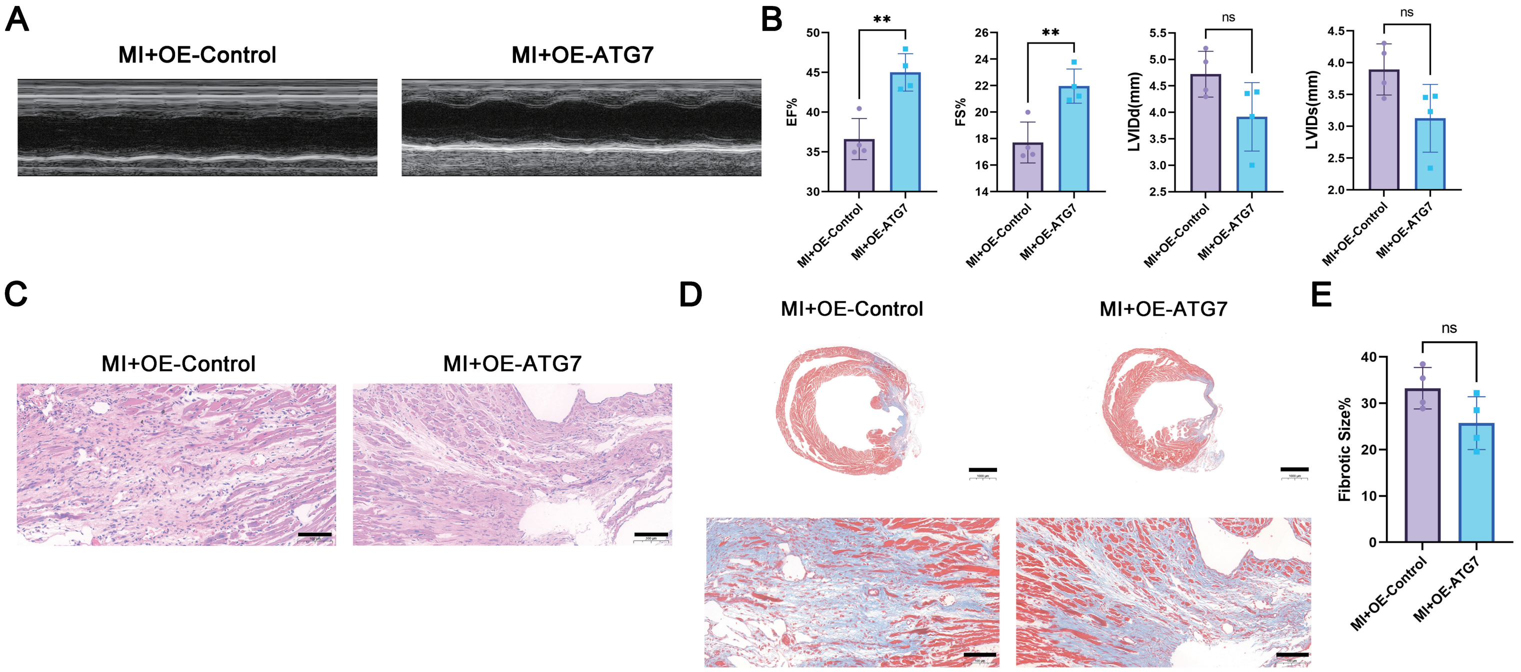

Figure S4. ATG7 overexpression regulated MI-induced heart injury in vivo.

(A) Representative photographs of M-mode echocardiography 1week post-MI (n=4). (B) Quantitative analysis of echocardiography. FS, fractional shortening; EF, ejection fraction; LVIDd, left ventricular internal diameter end-diastolic; LVIDs, left ventricular internal diameter end-systolic (n=4). (C) Hematoxylin and eosin (HE) staining of heart tissue sections 3 weeks post-MI (n=4). Scale bar: 50 µm. (D) Masson Trichrome staining of heart tissue sections 3 weeks post-MI (n=4). Scale bar: 1000 µm or 100 µm. (E) Percentage of left ventricle area occupied by scar tissue (n=4). All data were presented as mean±SD. ns represents $p > 0.05$, *$p < 0.05$, **$p < 0.01$, ***$p < 0.001$, ****$p < 0.0001$.
