# Supplementary material for: METTL3 regulates autophagy of hypoxia-induced cardiomyocytes by targeting ATG7
Source: Cell Death Discov. 2025 Feb 1;11:37. doi: 10.1038/s41420-025-02320-3 (PMC11787298; doi:10.1038/s41420-025-02320-3)
Supplement: Supplementary file 2 — Supplemental Material [file 41420_2025_2320_MOESM2_ESM.docx]

**Supplementary Table I. Antibodies used for western blot.**

| Antibodies | Concentration | Source | Manufacturer | Cat. number |
| --- | --- | --- | --- | --- |
| LC3B | 1:2000 | Rabbit | Abcam | ab192890 |
| P62 | 1:5000 | Rabbit | PTM BIO | PTM-6434 |
| ATG7 | 1:2000 | Rabbit | Abcam | ab133528 |
| c-caspase3 | 1:2000 | Rabbit | Cell Signaling Technology | 9664S |
| Caspase3 | 1:2000 | Rabbit | Cell Signaling Technology | 9662S |
| Bax | 1:2000 | Rabbit | Abcam | ab32503 |
| Bcl-2 | 1:2000 | Rabbit | Abcam | ab196495 |
| METTL3 | 1:1000 | Rabbit | Abcam | ab195352 |
| YTHDF2 | 1:2000 | Rabbit | Proteintech | 24744-1-AP |
| ATG5 | 1:2000 | Rabbit | Proteintech | 10181-2-AP |
| Beclin-1 | 1:2000 | Rabbit | Proteintech | 11306-1-AP |
| β-actin | 1:10000 | Mouse | Proteintech | HRP-60008 |
| Goat Anti-Rabbit IgG H&L | 1:10000 | Goat | Abcam | ab6721 |

**Supplementary Table II. Primer list.**

| Gene | Primer Sequence |
| --- | --- |
| METTL3 | 5’-CACGCTGCCTCAGATGTTGACC-3’  5’-CTGACCTTCTTGCTCTGCTGTTCC-3’ |
| YTHDF1 | 5’-CTCTGTTGGAAGTGCTCAGCCTAC-3’  5’-GTACTTGATGGAGCGGTGGATGTC-3’ |
| YTHDF2 | 5’-CAGACACAGCCATTGCCTCCAC-3’  5’-CCGCCTGAGACTGTCCTACTCC-3’ |
| YTHDF3 | 5’-CAGCCTTGAGCAGCAGTGGTATG-3’  5’-AGGAGGTGGTGGCACAGCAG-3’ |
| ULK1 | 5’-TACACAGCAAGGGCATCATTCACC-3’  5’-CGGGCAAATCCAAAGTCAGCAATC-3’ |
| ATG3 | 5’-TACACAGCAAGGGCATCATTCACC-3’  5’-GAGGTGGTGGGAGGTGAGGATG-3’ |
| ATG4b | 5’-ACTTCAGTGTCCTCAACGCTTTCC-3’  5’-TGCCTTCGCCAACTCCCATTTG-3’ |
| ATG4d | 5’-TGACATCCAGCGGTTTCAAAGGG-3’  5’-GCCAGCATCATCTGACCACTTCG-3’ |
| ATG5 | 5’-ATATGAAGGCACACCCCTGAAATGG-3’  5’-GTGATGTTCCAAGGCAGAGCTGAG-3’ |
| ATG7 | 5’-GATGGTGAACCTCAGCGGATGTATG-3’  5’-CAGCAGCAGGCACTTGACAGAC-3’ |
| ATG10 | 5’-TAGGCGATGGCTGGGAATGGAG-3’  5’-CCGATGTGGTGTTGGAGTCTCTTTC-3’ |
| ATG12 | 5’-TCTCCCCAGAAACAGCCATCCC-3’  5’-AGTGTCTCCTACAGCCTTCAGCAG-3’ |
| ATG13 | 5’-ACCGATTGTCACTGCTGCTGAAG-3’  5’-TGCCCTTGCTTCCTGGAGAGTC-3’ |
| ATG14 | 5’-AAGGAGAAGATTCAGCGGCACAAC-3’  5’-CATTGGGAAGATGACAGAGGTGAGC-3’ |
| ATG16L1 | 5’-CAAGCCGAATCTGGACTGTGGATG-3’  5’-CAGCAGGAACTTGGCAGAGAGAAC-3’ |
| BECN1 | 5’-TCAAGATCCTGGACCGAGTGACC-3’  5’-CTCCTCTCCTGAGTTAGCCTCTTCC-3’ |
| ZKSCAN3 | 5’-CCAGTGAGCAGGAAGGCAAGTTAC-3’  5’-CCCACATTCACGGCAGTAGAACC-3’ |
| BRD4 | 5’-CCAAGATGCCTGATGAGCCTGAAG-3’  5’-GAGGAACTATCACTGCTGCTGTCAC-3’ |
| TFEB | 5’-GGTGCAGTCCTACCTGGAGA-3’  5’-GTGGGCAGCAAACT TGTTCC-3’ |

**Supplementary Table III. Antibodies used for immunofluorescent staining.**

| Antibodies | Concentration | Source | Manufacturer | Cat. number |
| --- | --- | --- | --- | --- |
| METTL3 | 1:1000 | Rabbit | Abcam | ab195352 |
| LC3B | 1 µg/ml | Rabbit | Abcam | ab192890 |
| ATG7 | 1:500 | Rabbit | Abcam | ab133528 |
