# Supplementary material for: METTL3 regulates autophagy of hypoxia-induced cardiomyocytes by targeting ATG7
Source: Cell Death Discov. 2025 Feb 1;11:37. doi: 10.1038/s41420-025-02320-3 (PMC11787298; doi:10.1038/s41420-025-02320-3)

Figure 1A

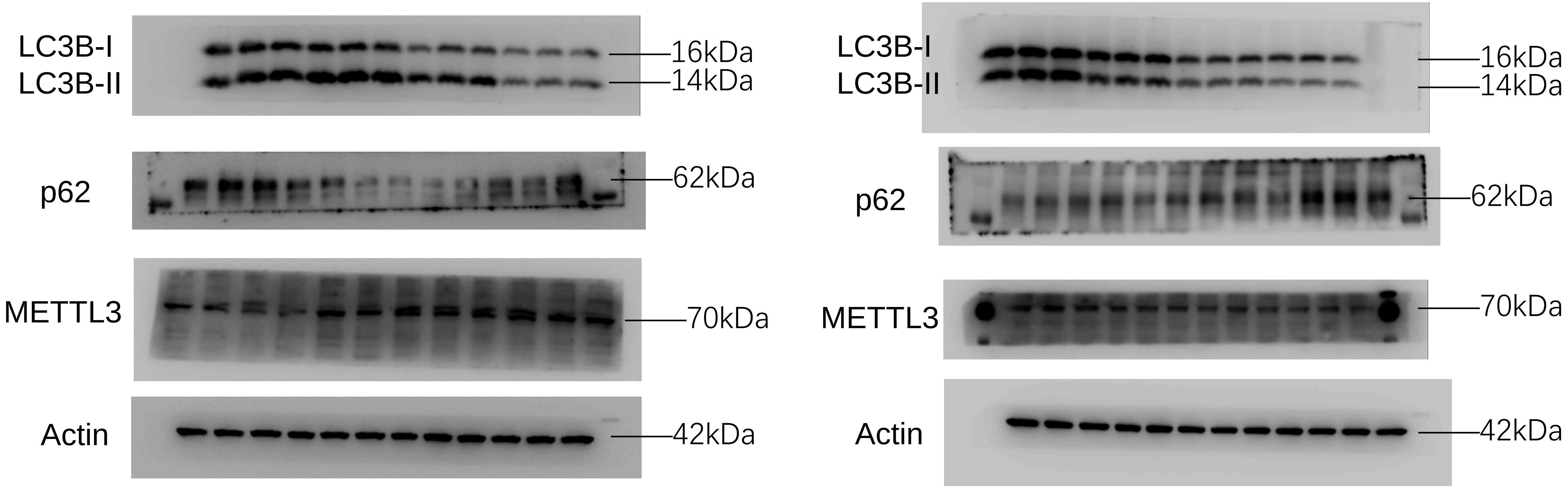

LC3B-I
LC3B-II
16kDa
14kDa
p62
62kDa
METTL3
70kDa
Actin
42kDa
LC3B-I
LC3B-II
16kDa
14kDa
p62
62kDa
METTL3
70kDa
Actin
42kDa

Figure 1D

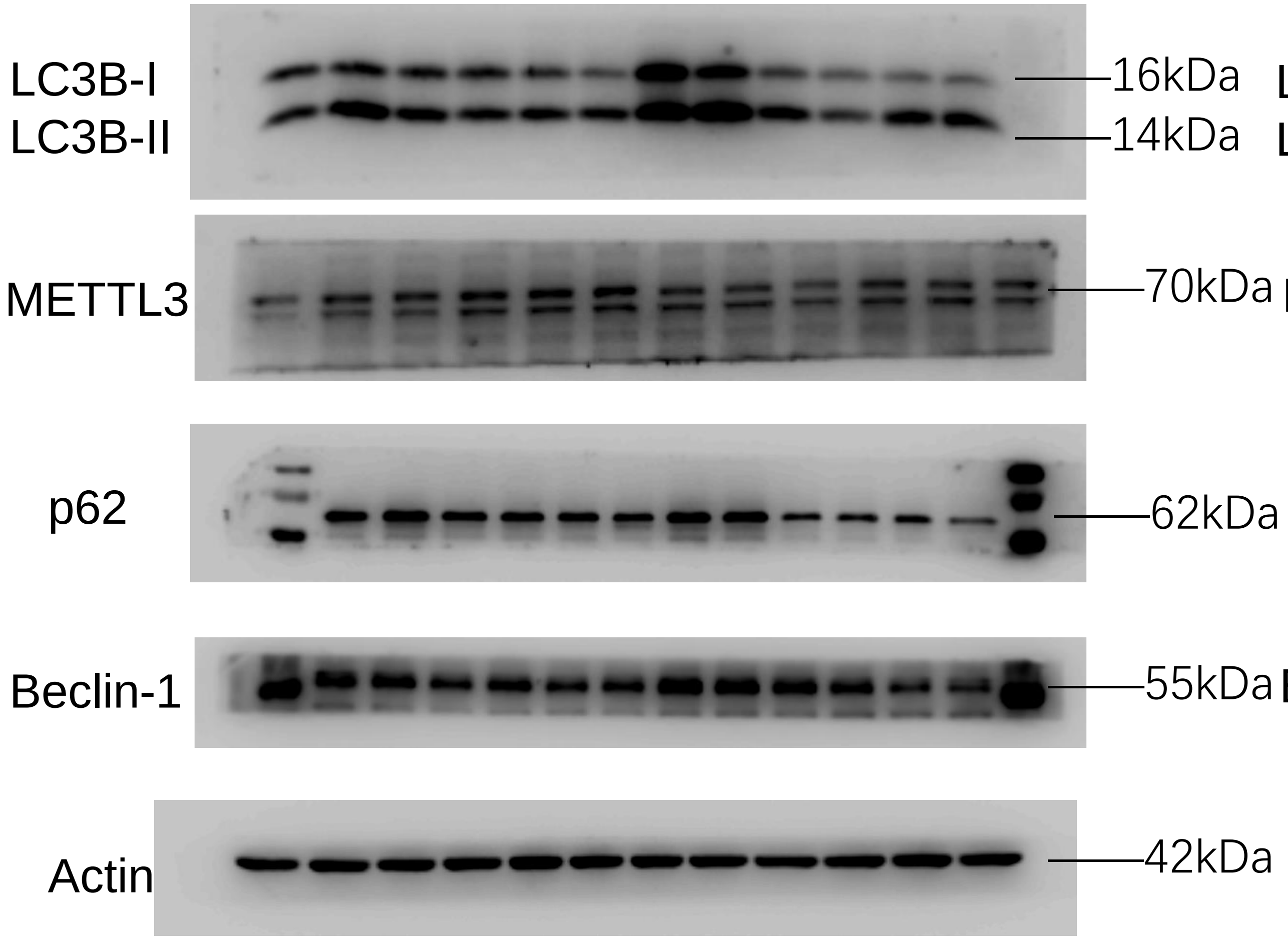

Figure 1E

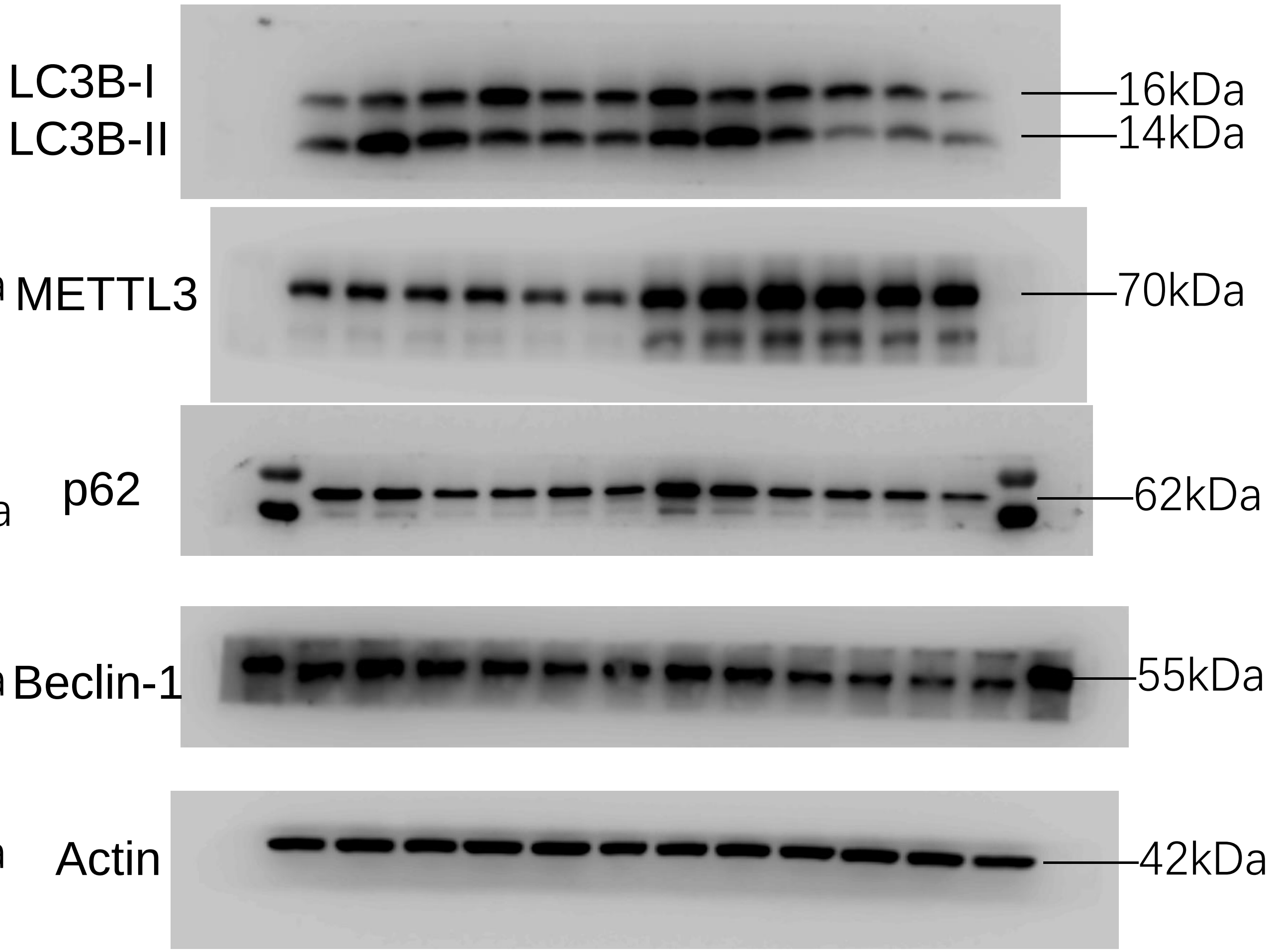

Figure 2A

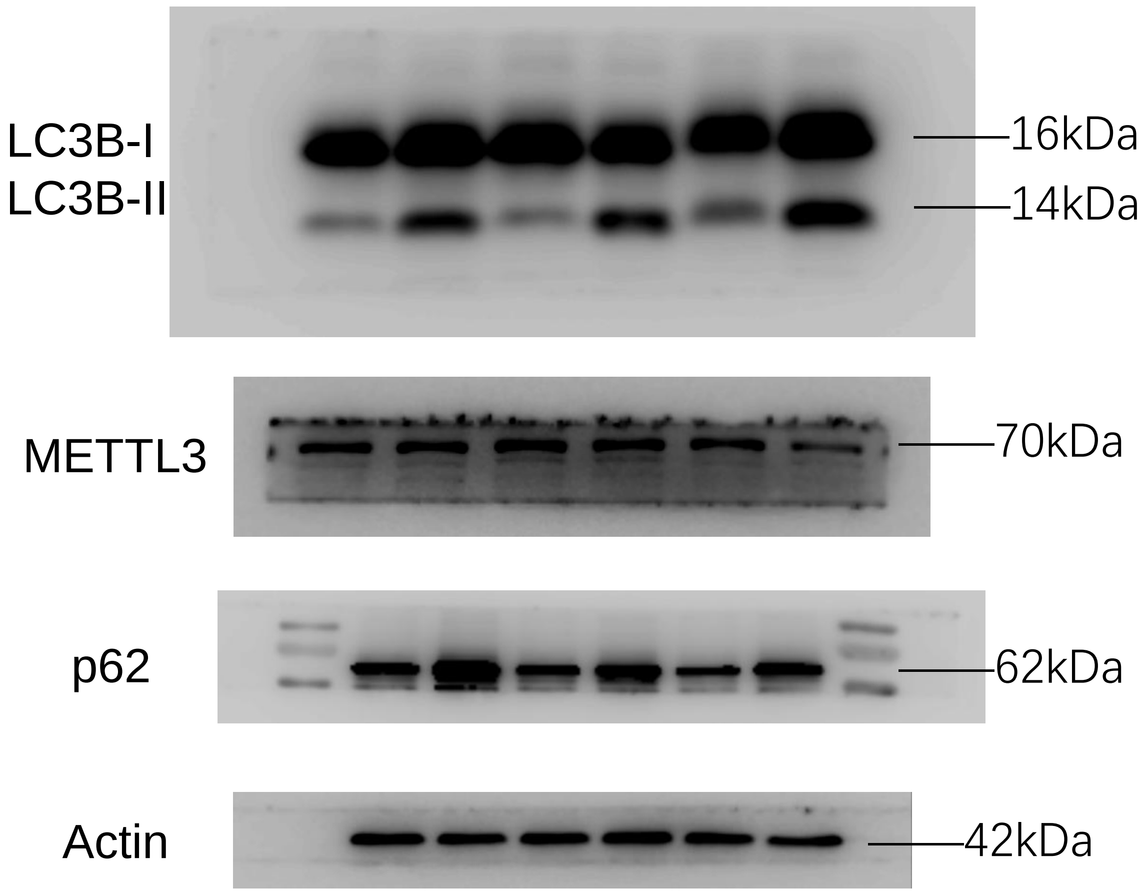

Supplementary Figure 1E

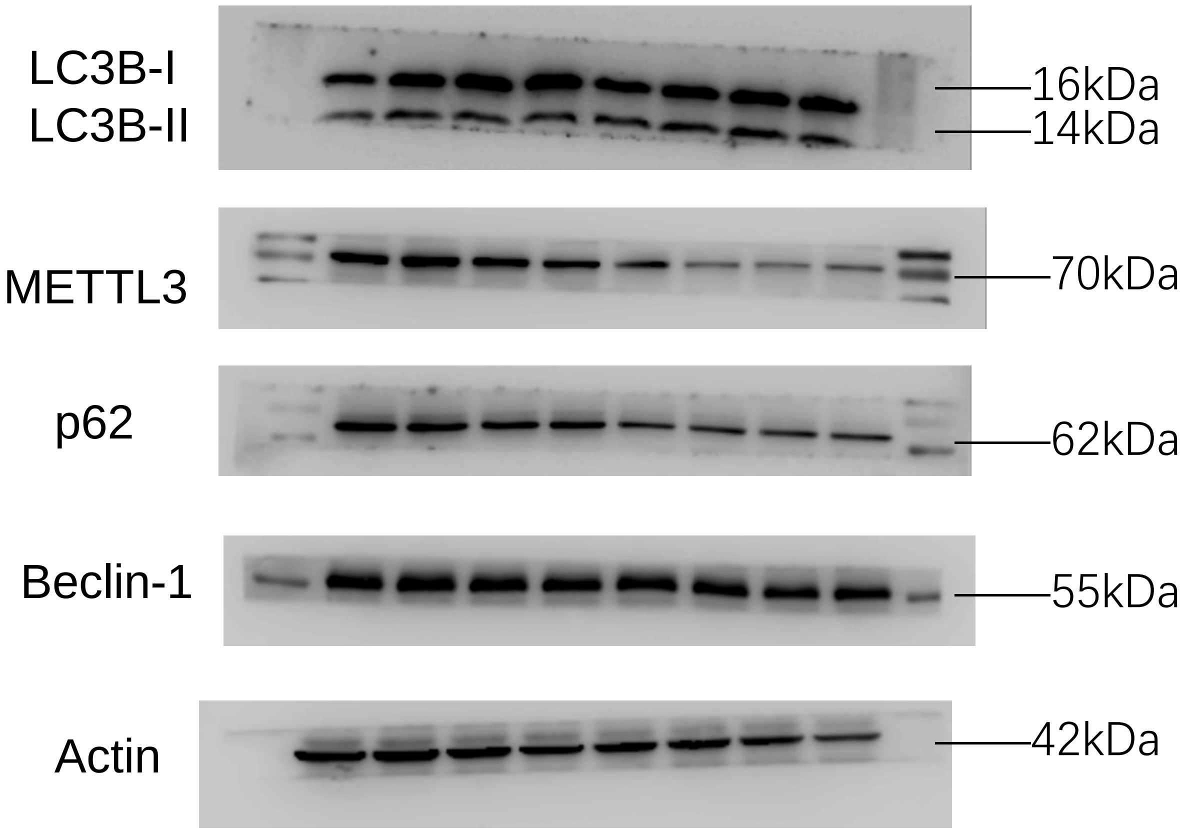

Figure 2E

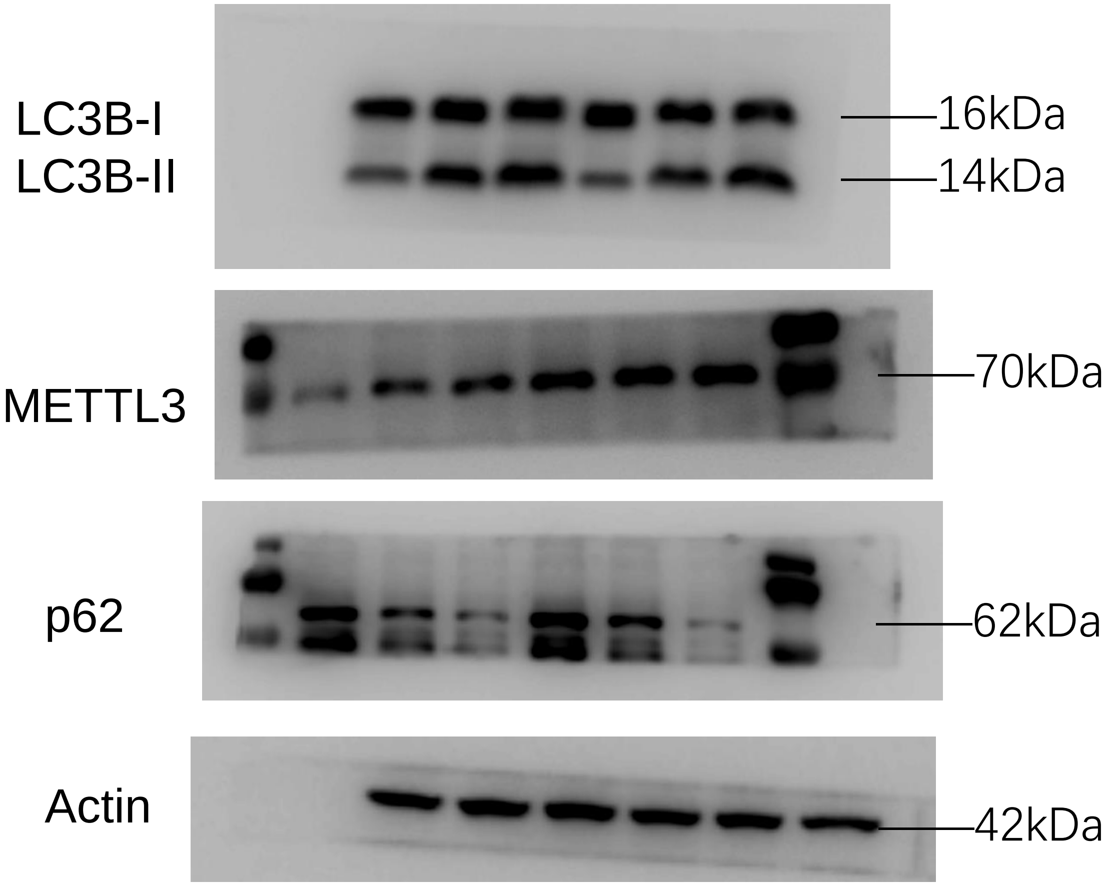

Figure 2J

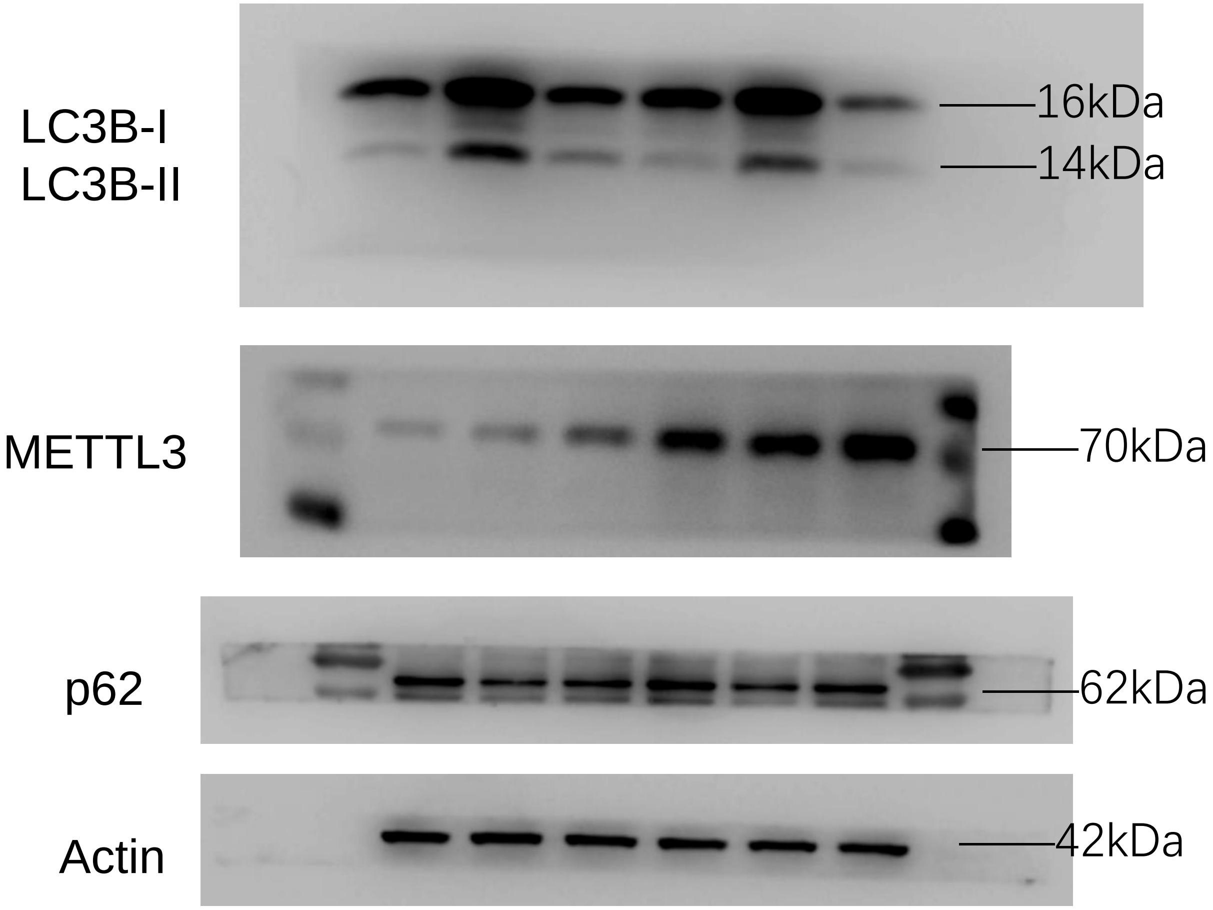

Figure 2G

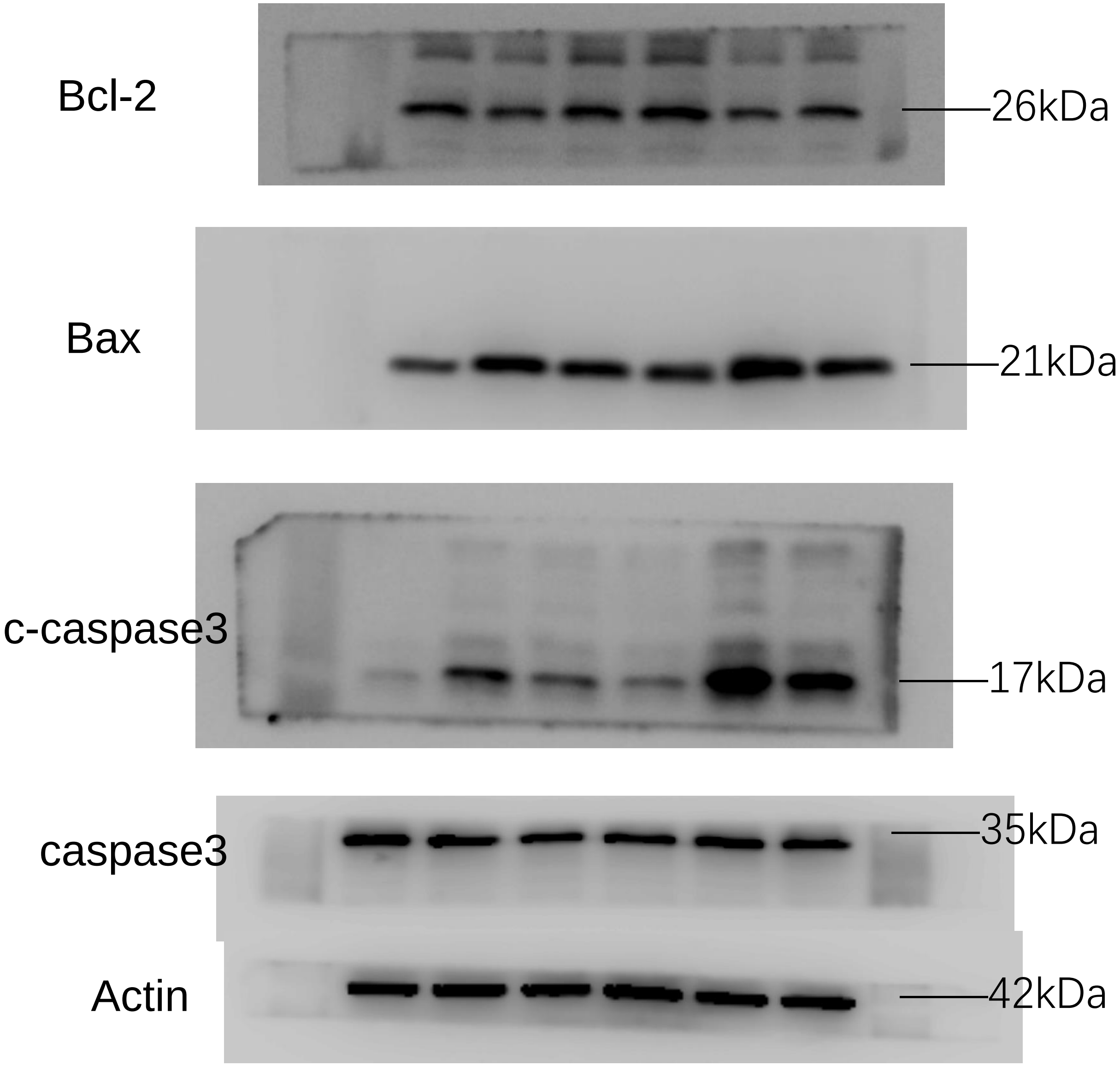

Figure 2L

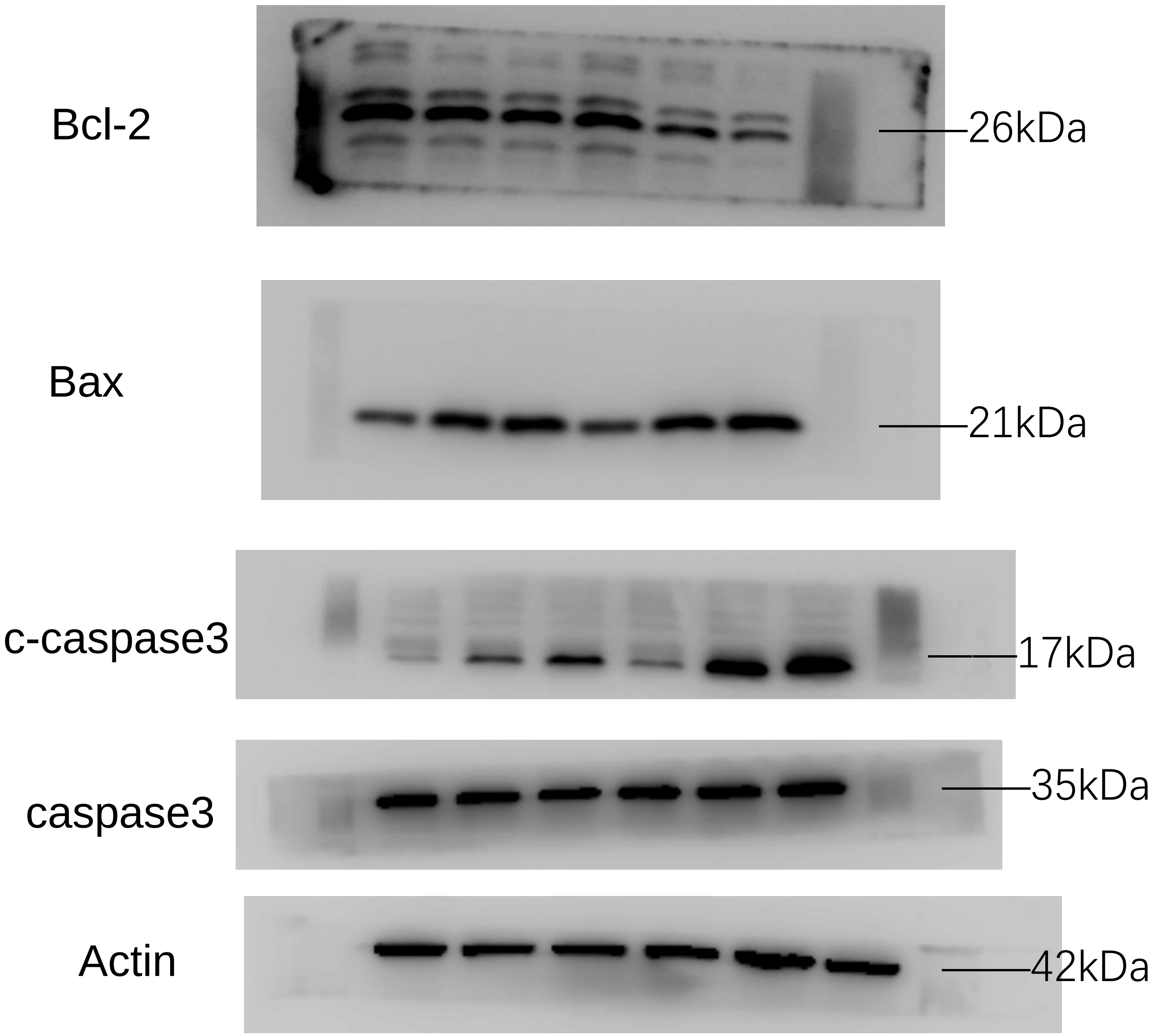

Figure 3G

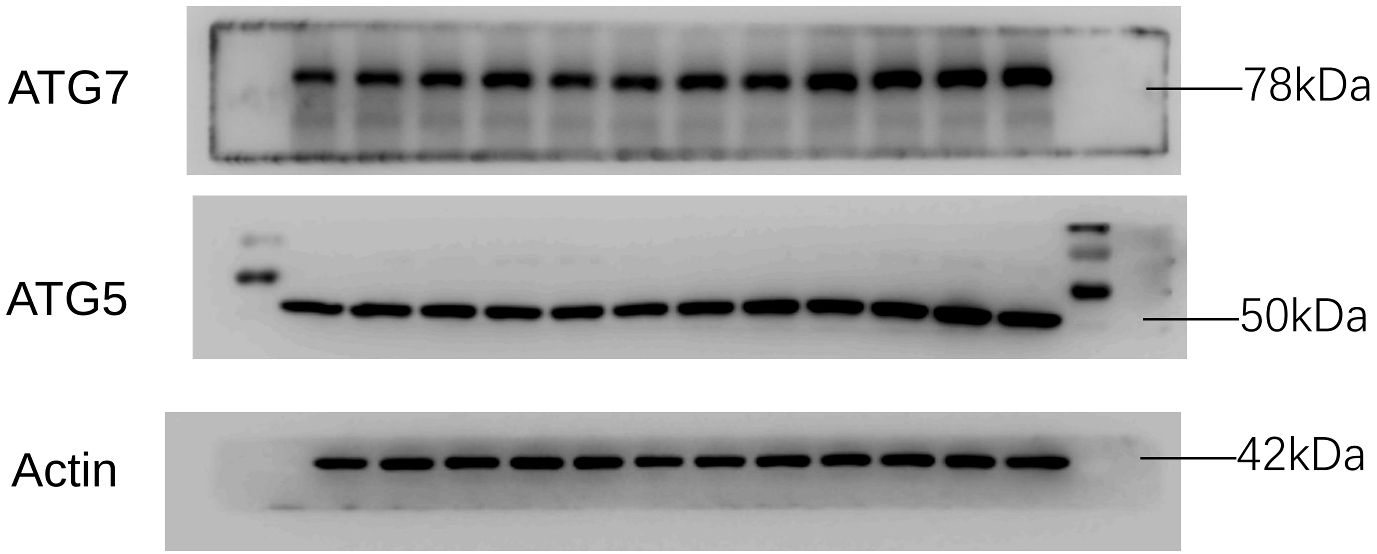

Supplementary Figure 3C

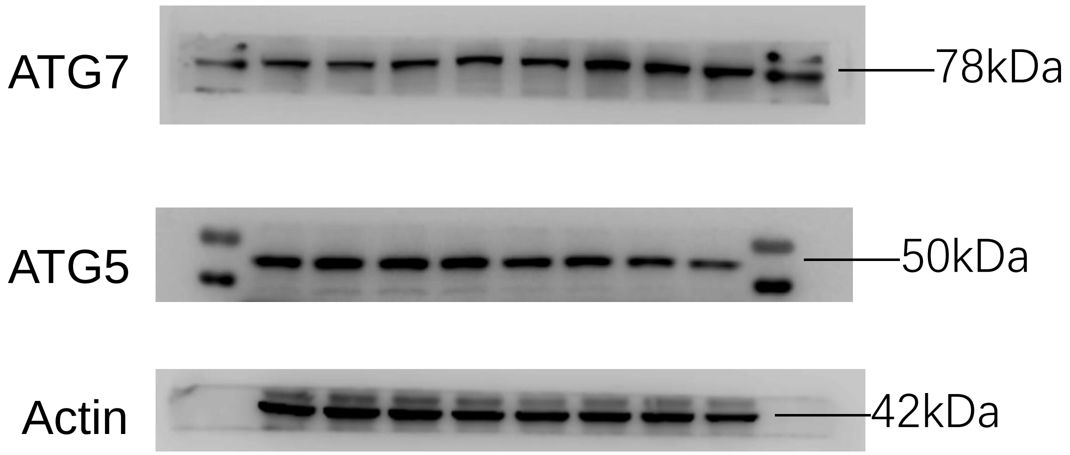

Figure 3H

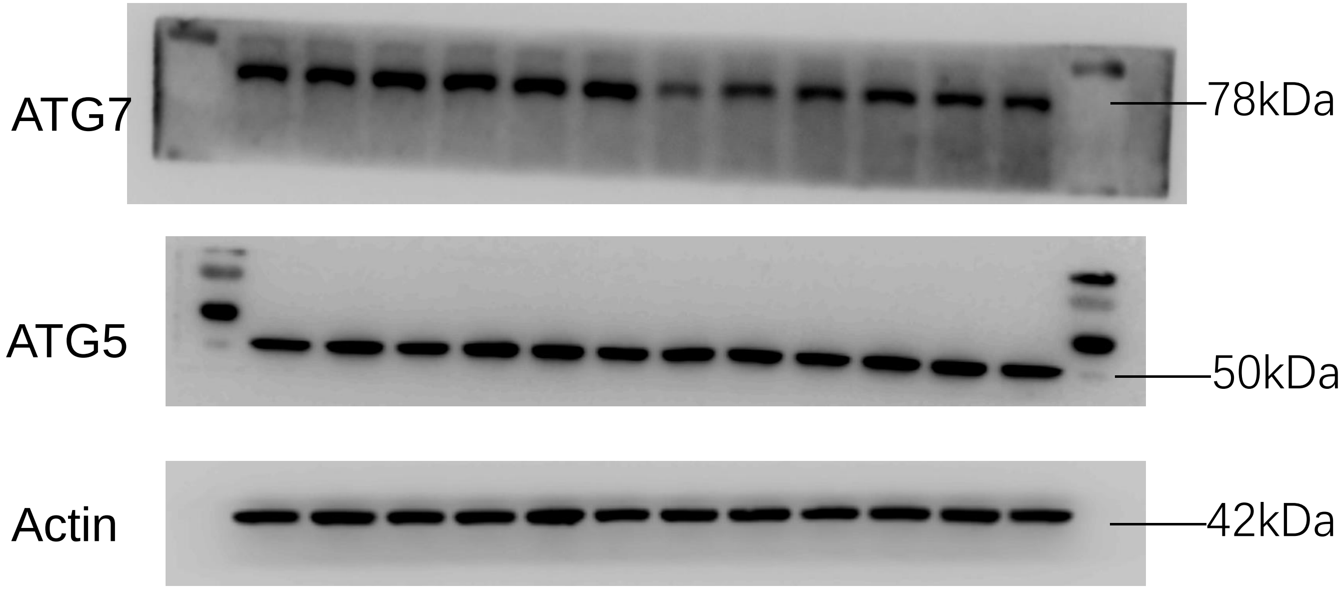

Figure 3I

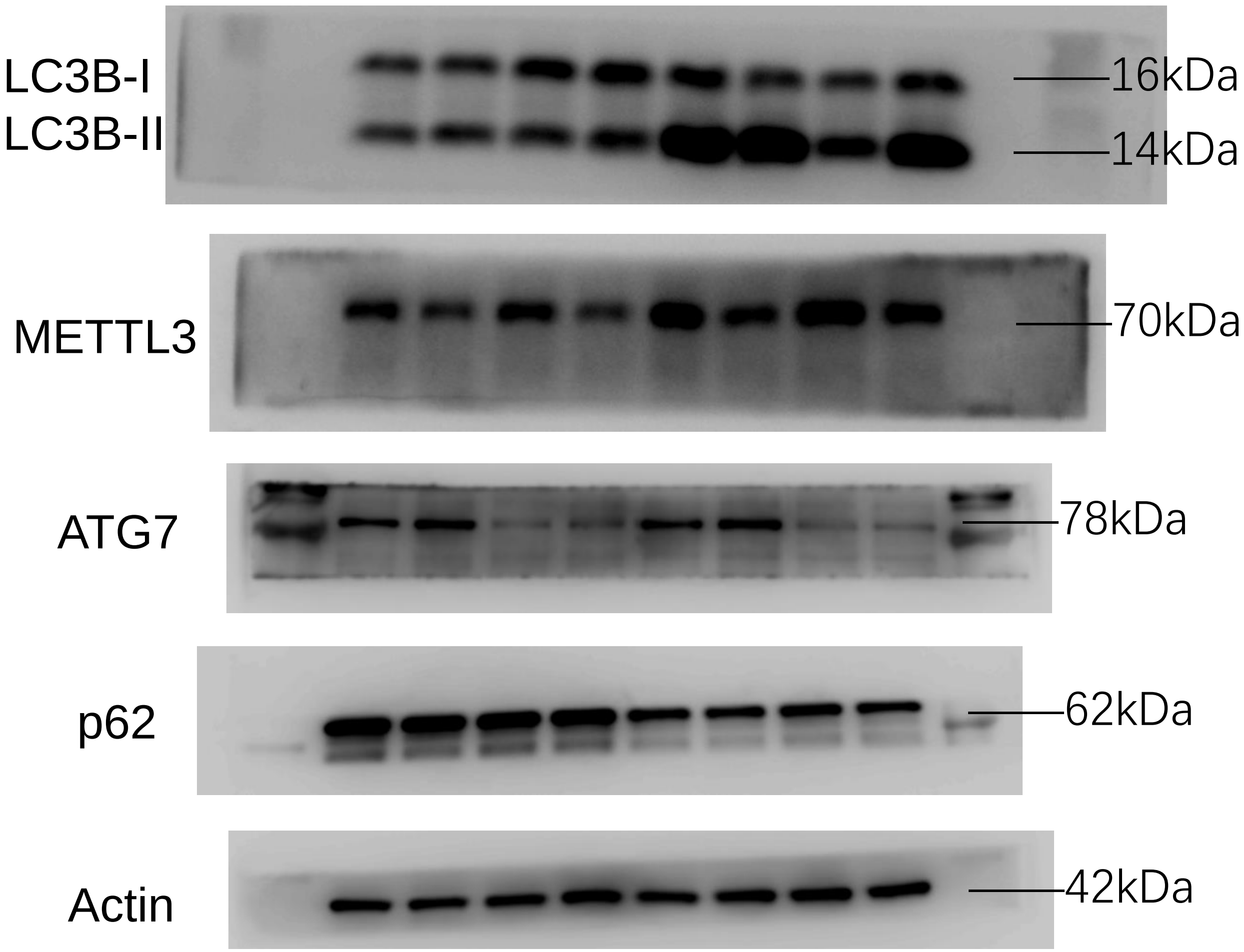

Figure 3K

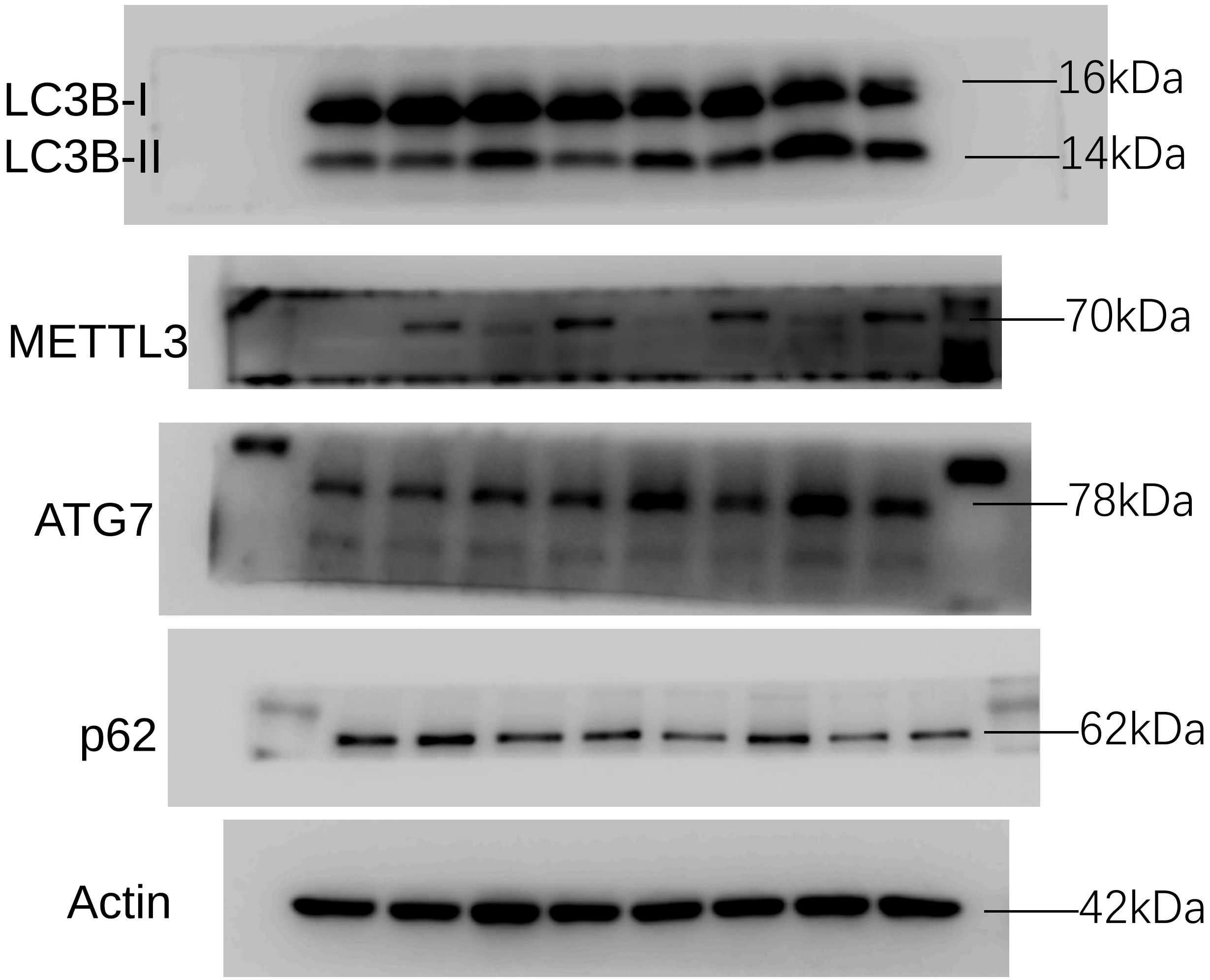

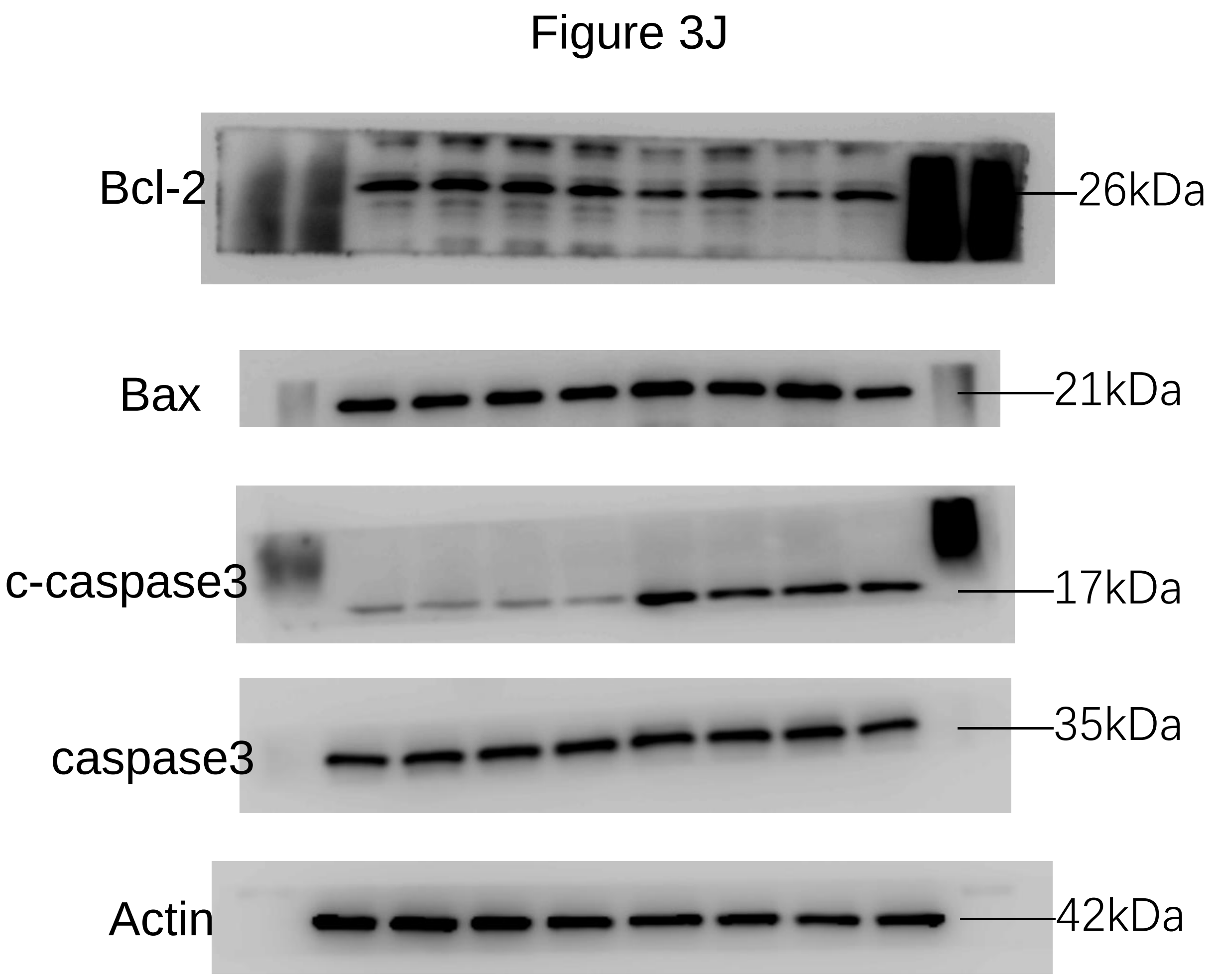
Figure 3J
Bcl-2
26kDa
Bax
21kDa
c-caspase3
17kDa
caspase3
35kDa
Actin
42kDa

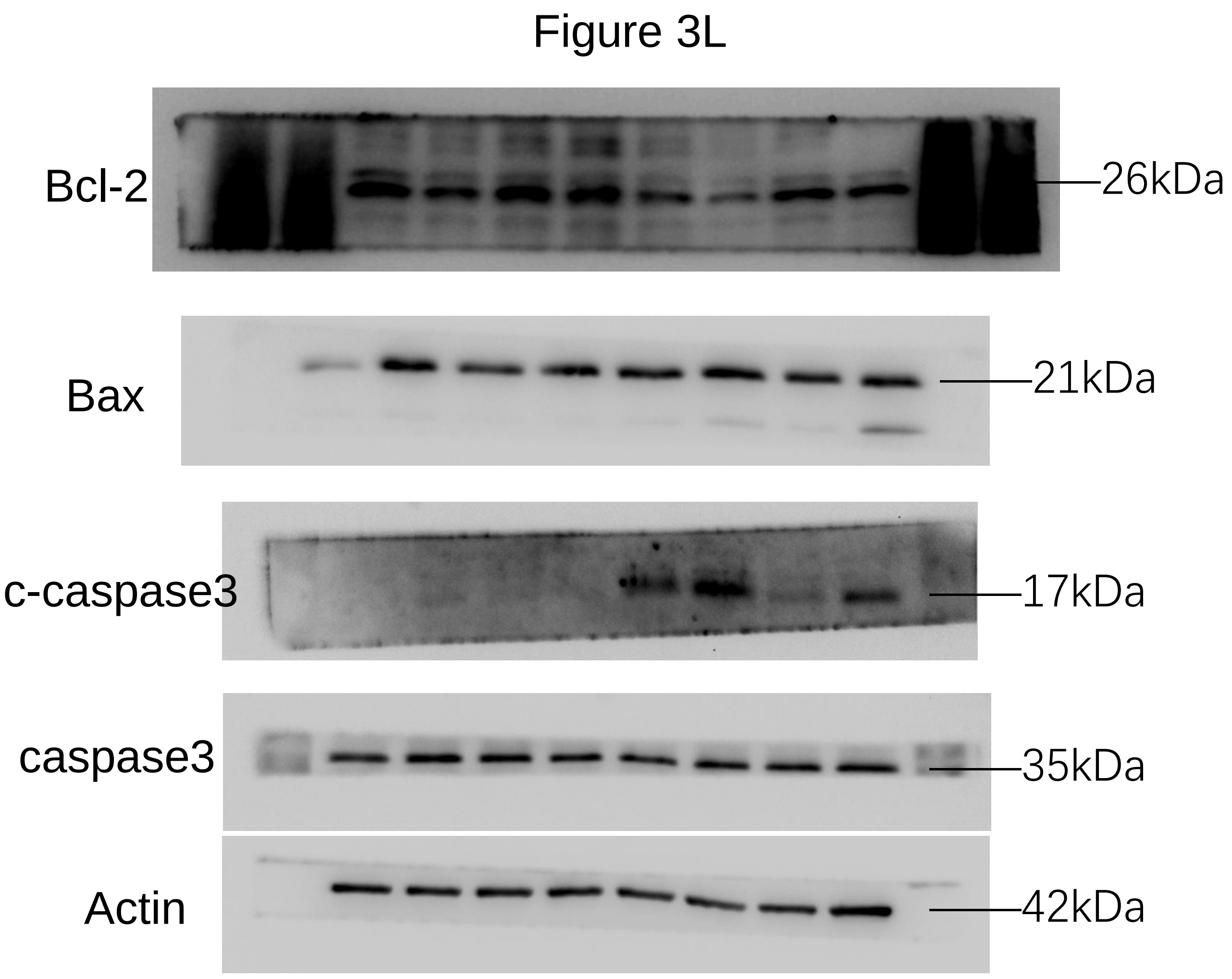
Figure 3L
Bcl-2
26kDa
Bax
21kDa
c-caspase3
17kDa
caspase3
35kDa
Actin
42kDa

Figure 5B

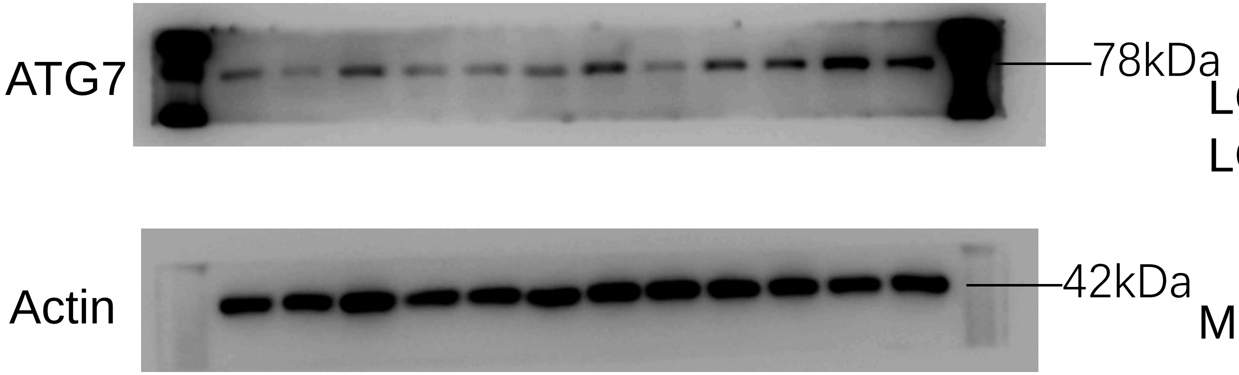

Figure 5E

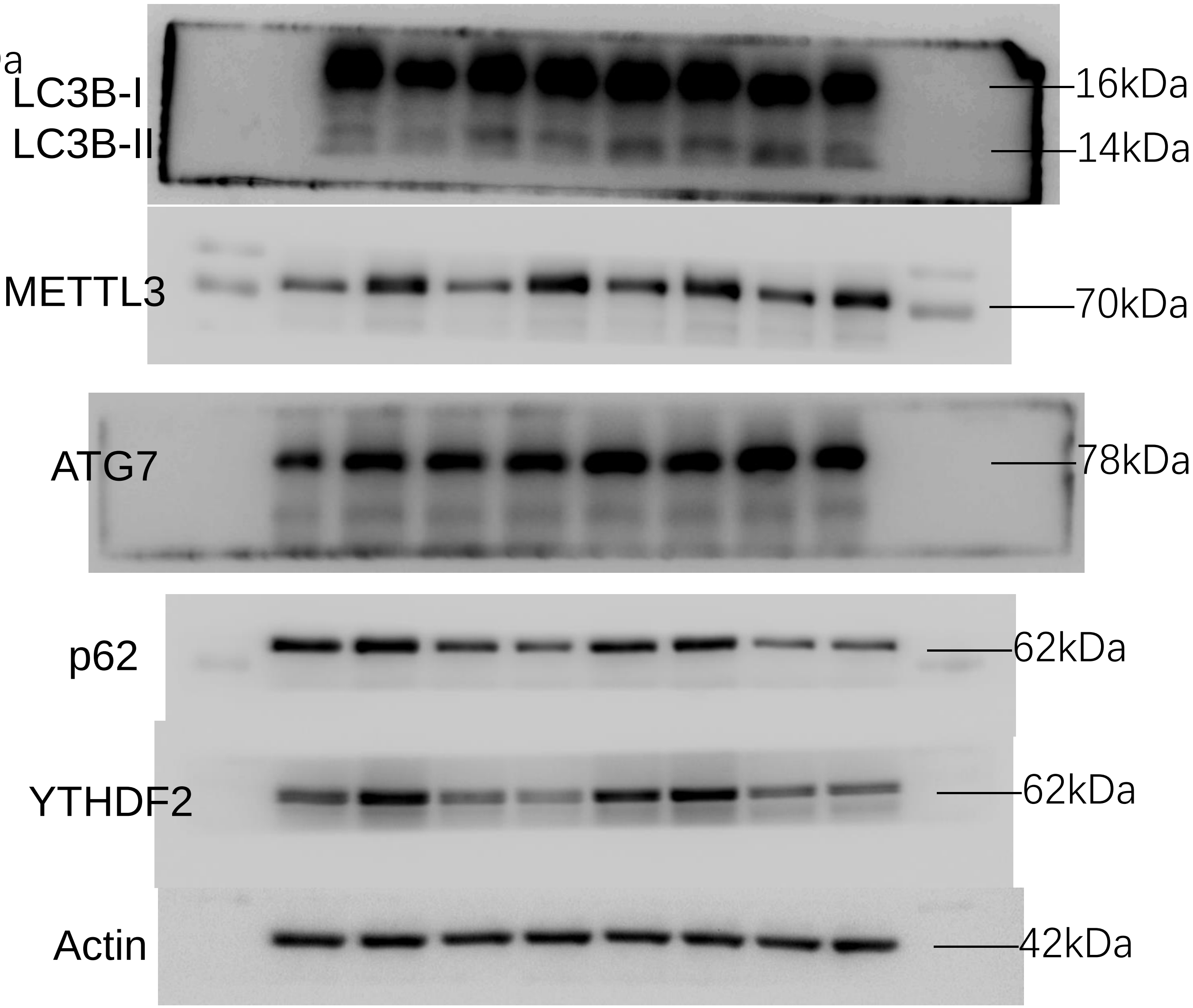

Figure 6A

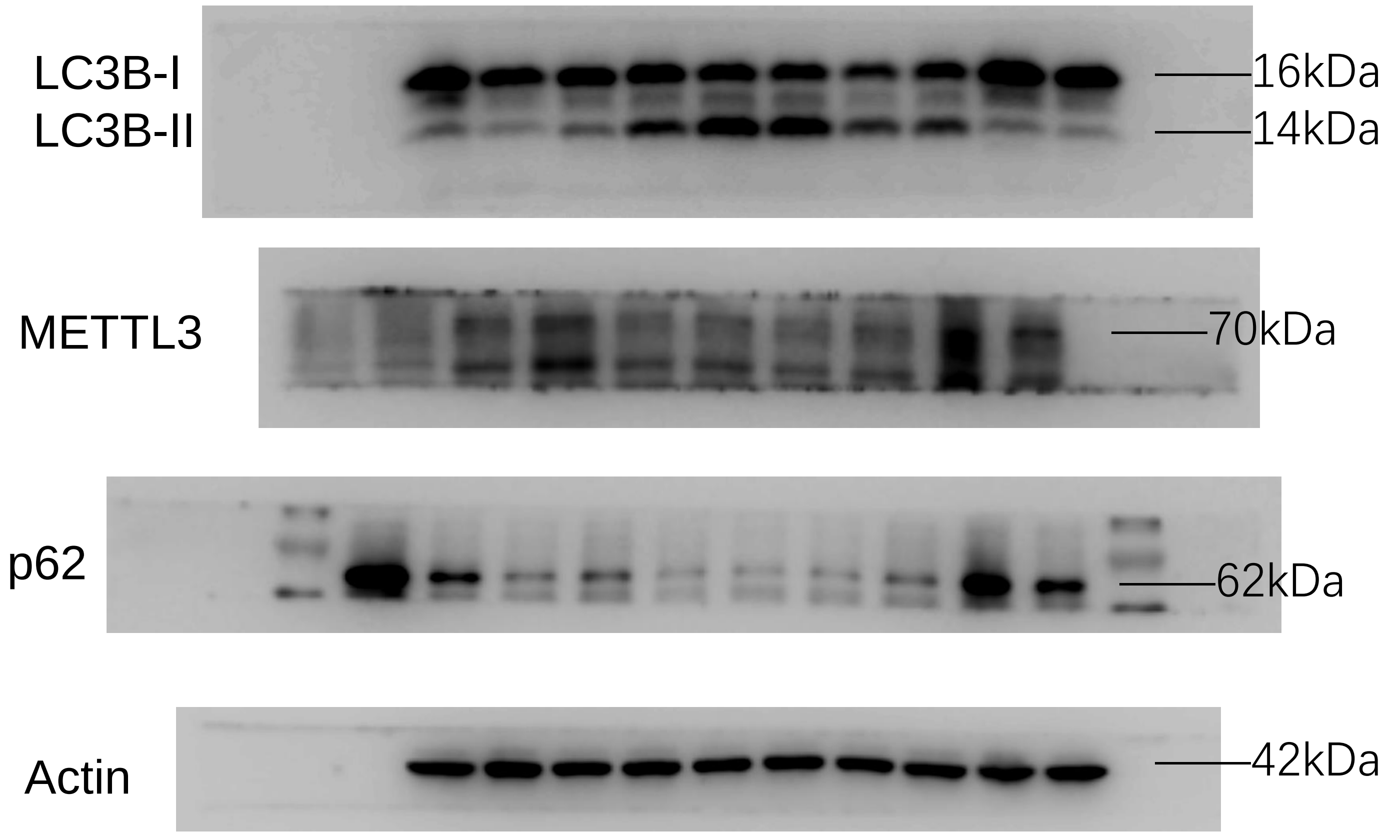

Figure 6C

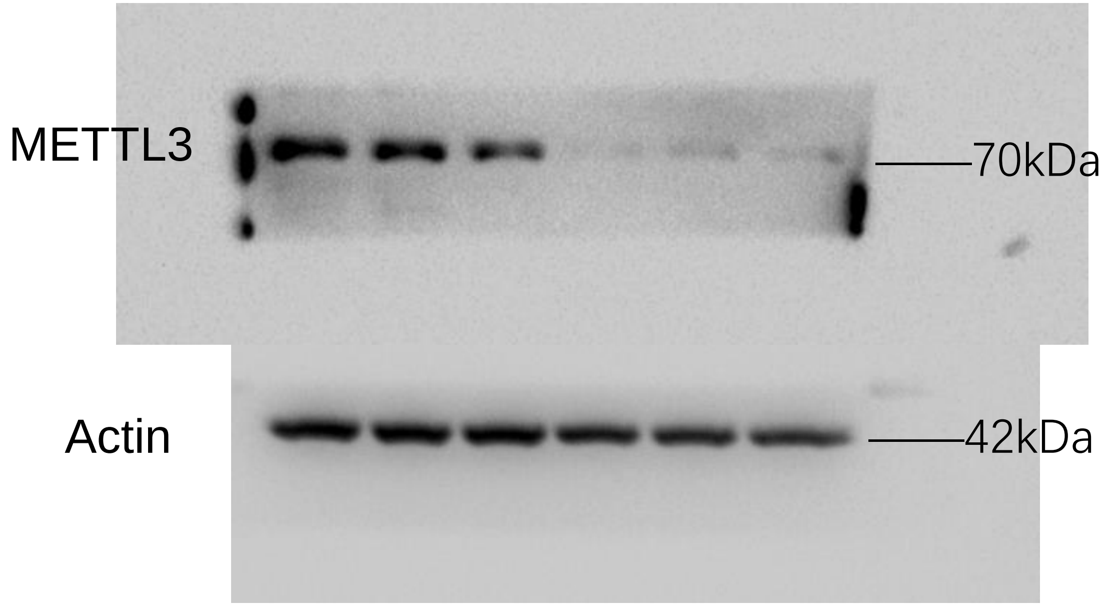

Figure 6D

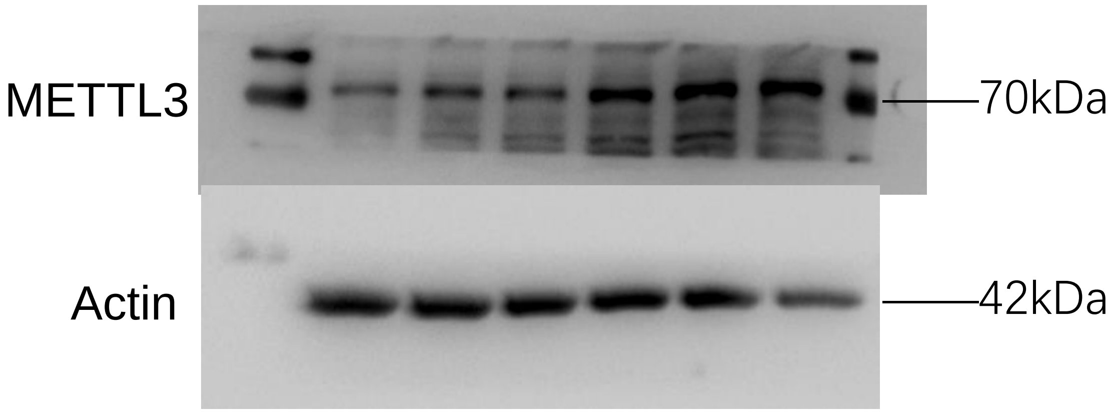

Figure 6G

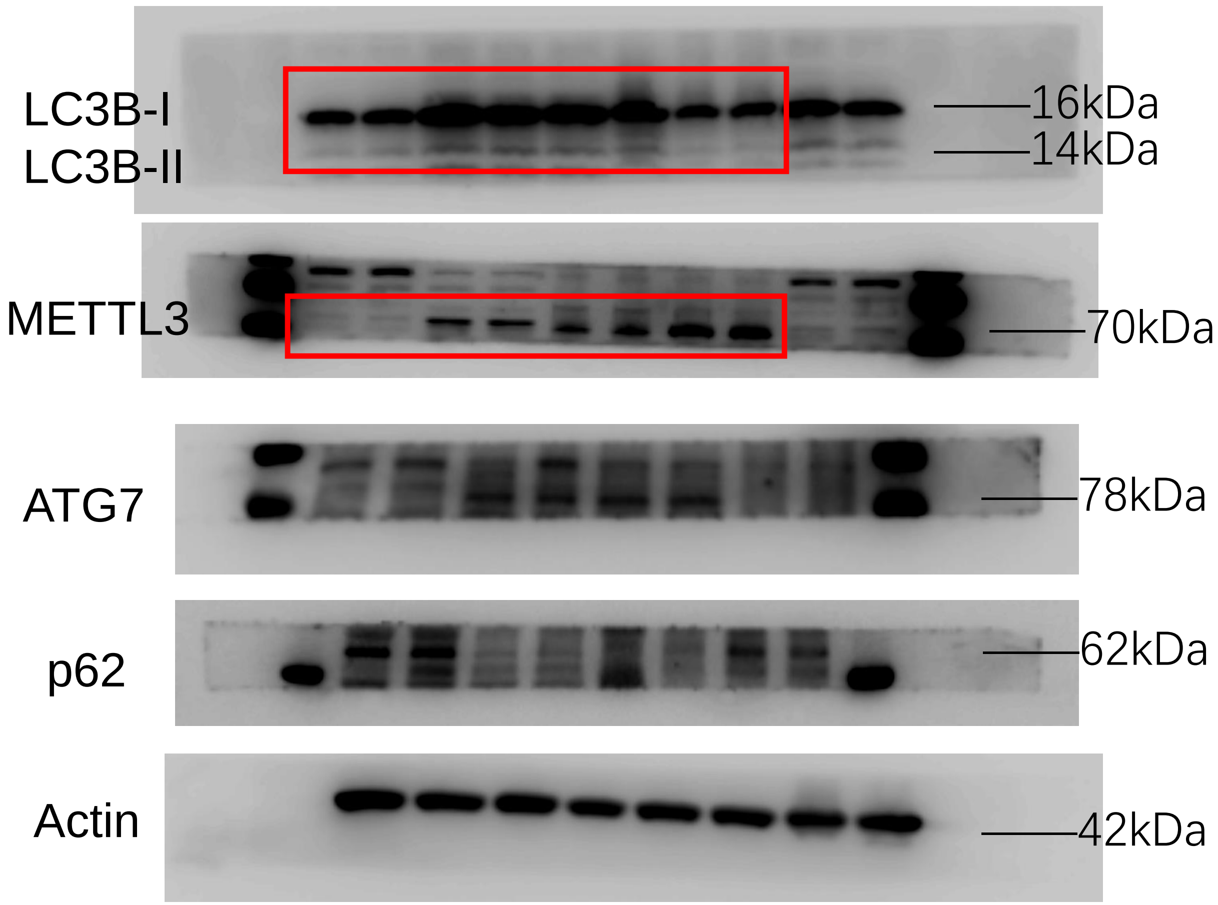

Figure 6J

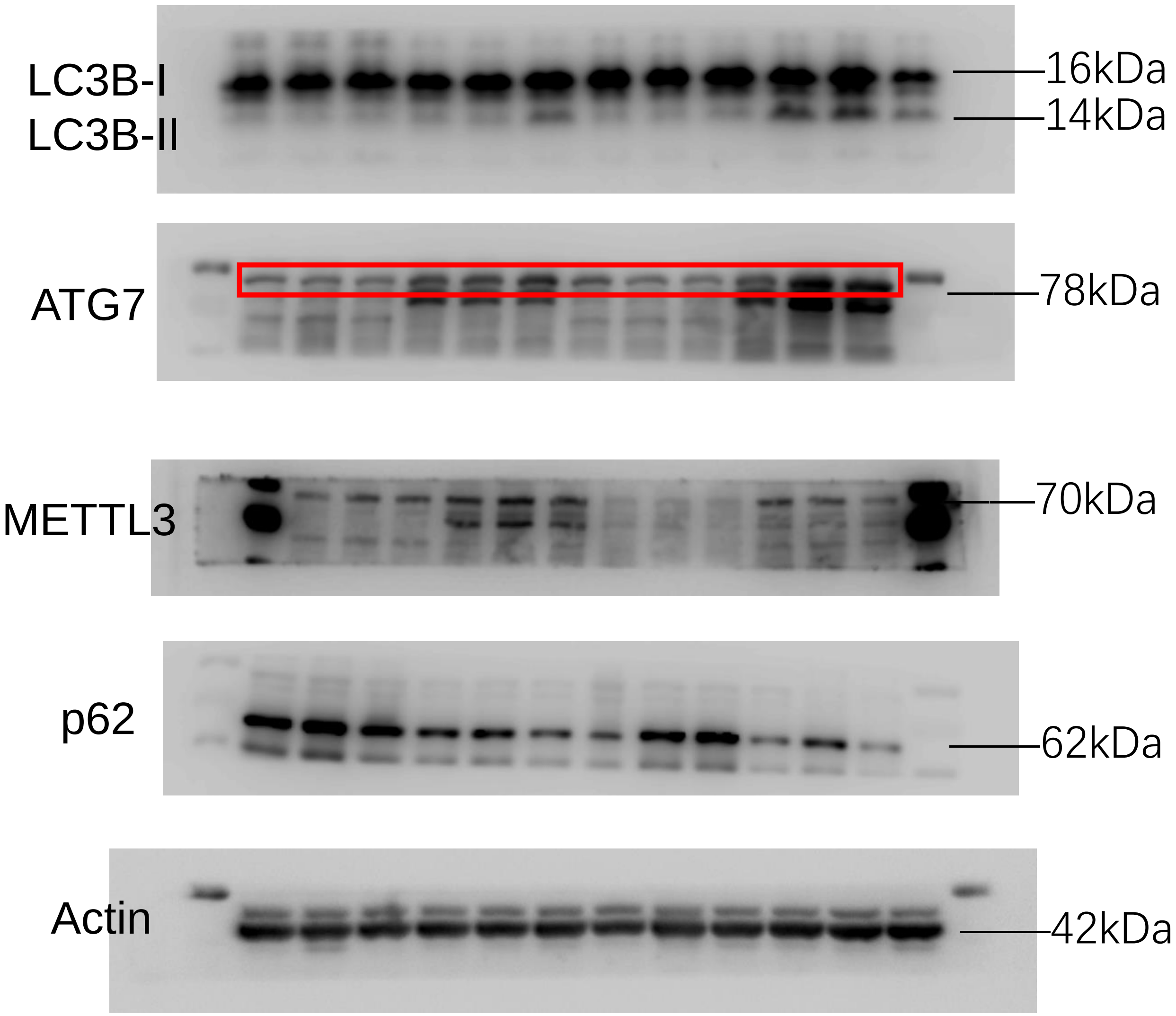

Figure 7A

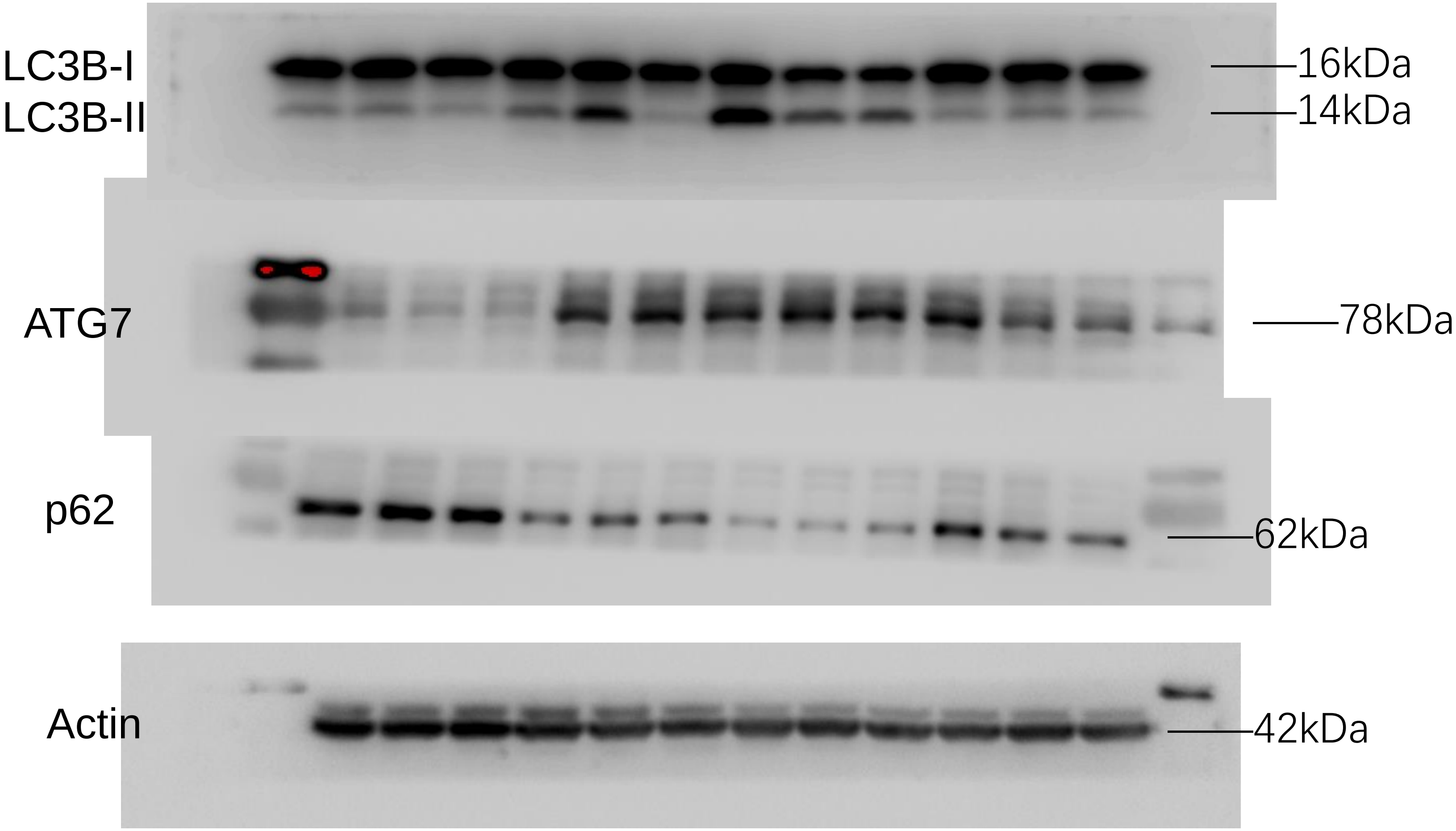

Supplementary Figure 1A

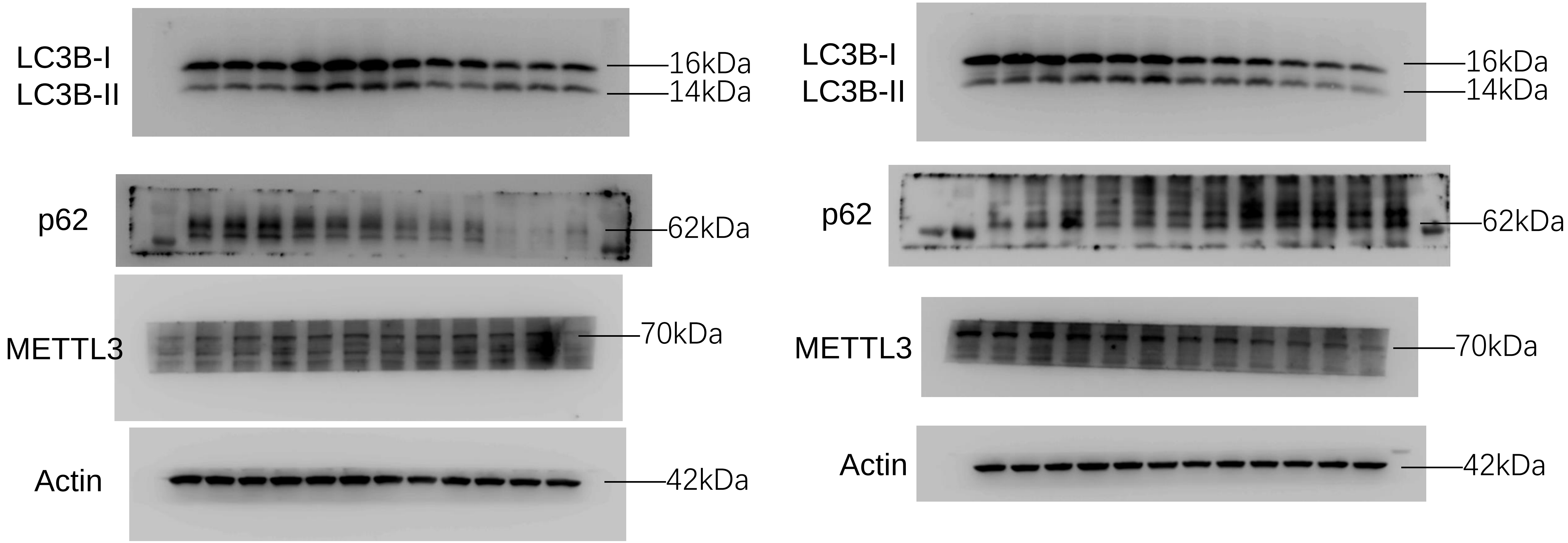

Supplementary Figure 1C

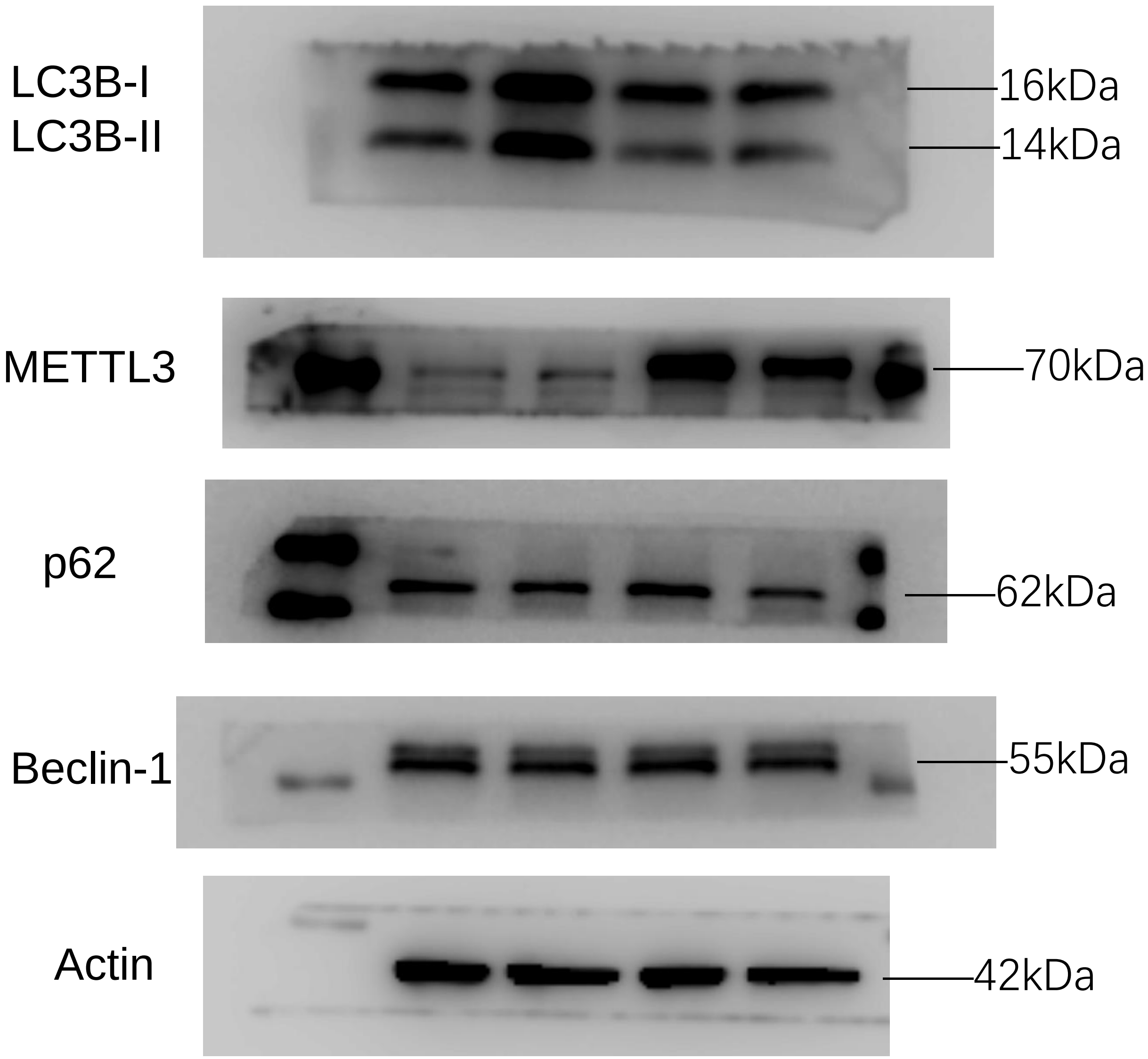

Supplementary Figure 1D

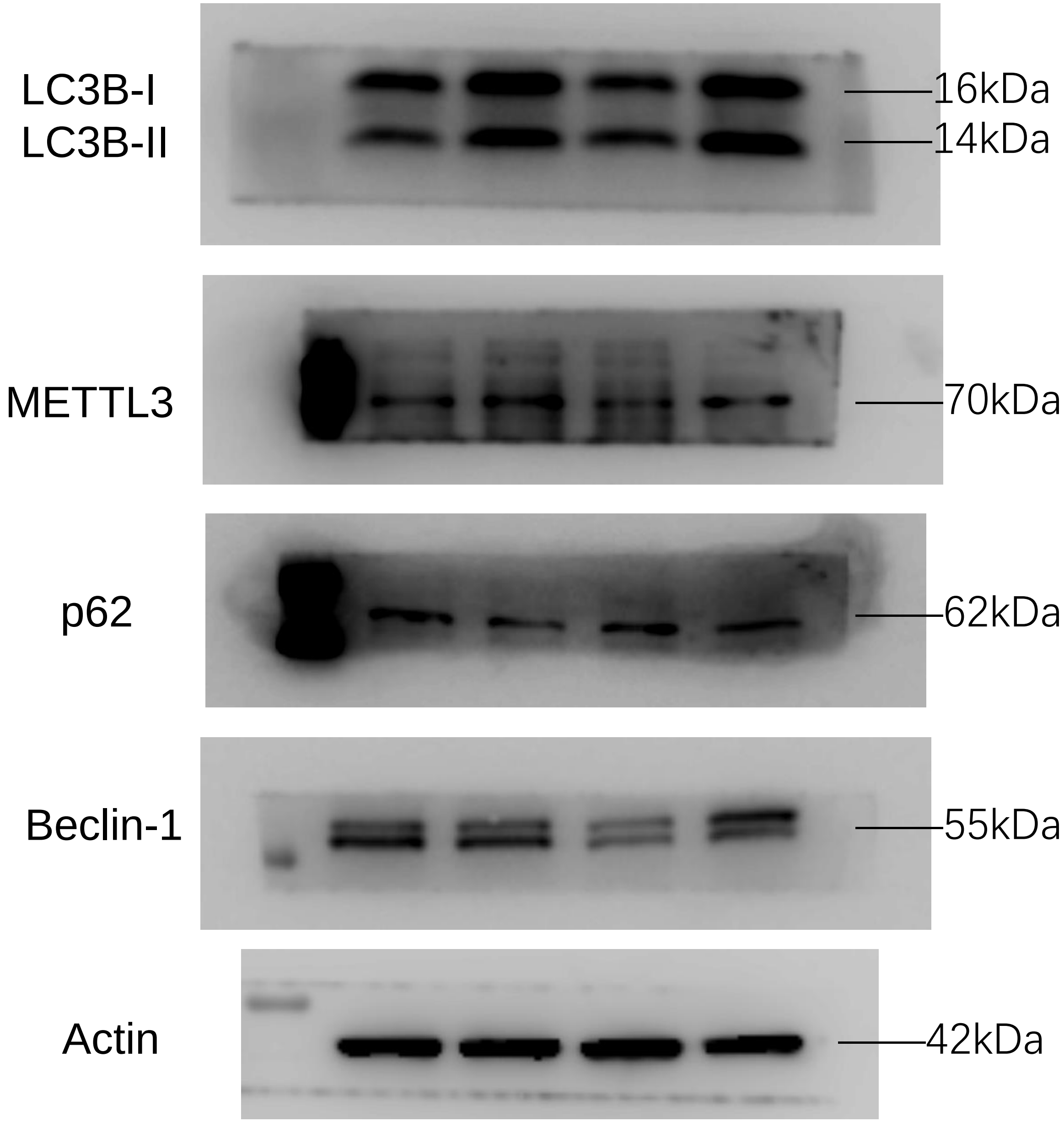

Supplementary Figure 2A

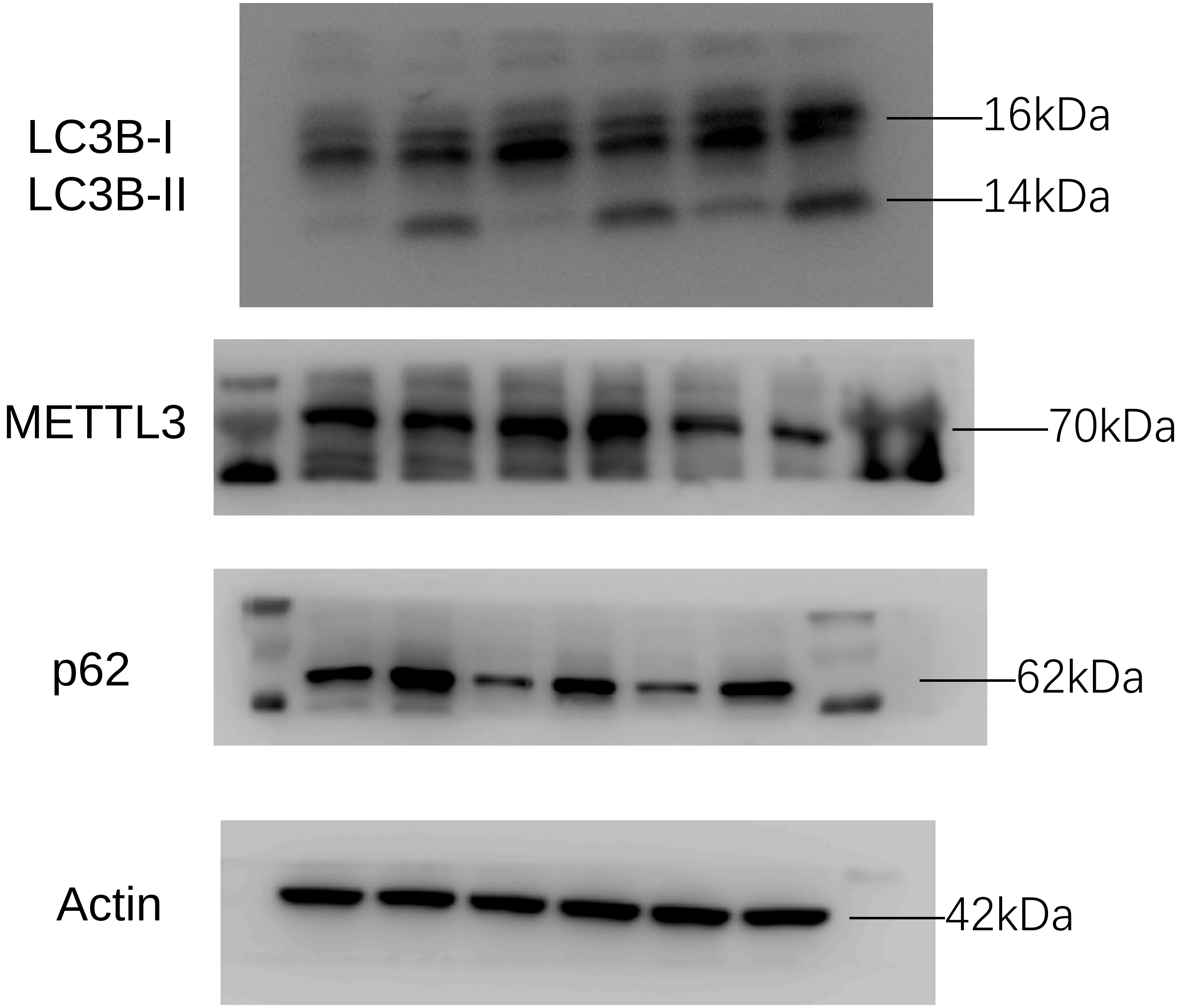

Supplementary Figure 3A

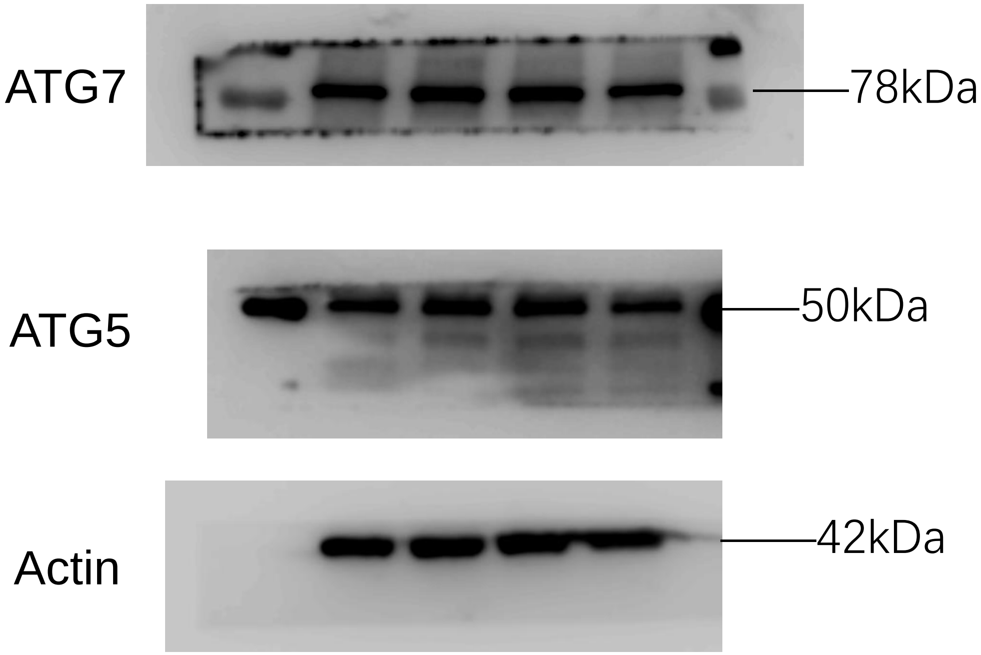

Supplementary Figure 3B

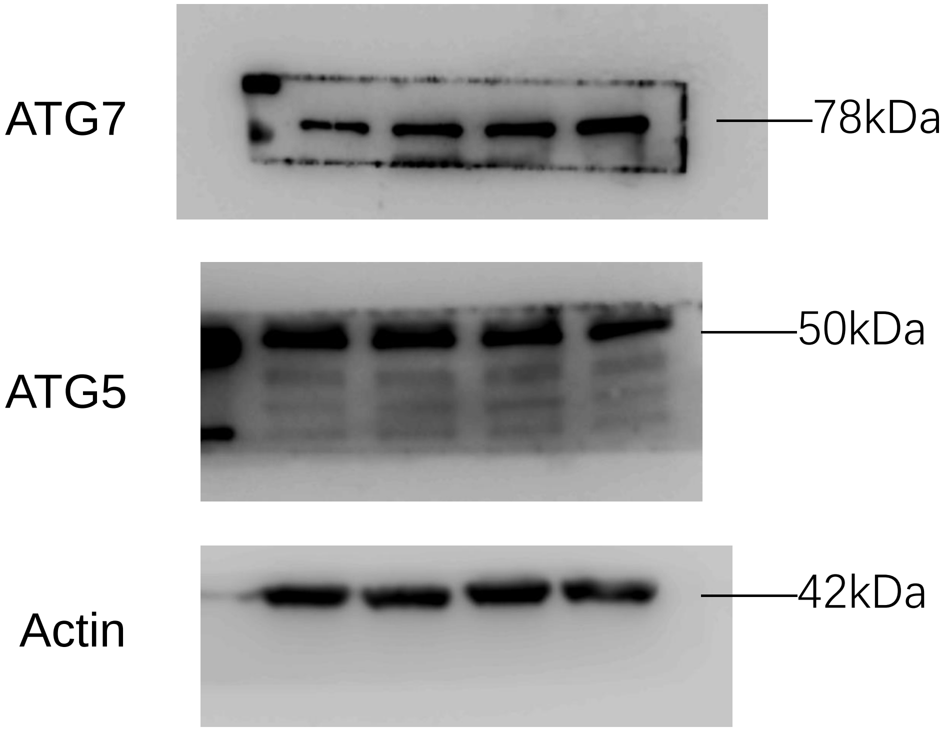

Supplementary Figure 3D

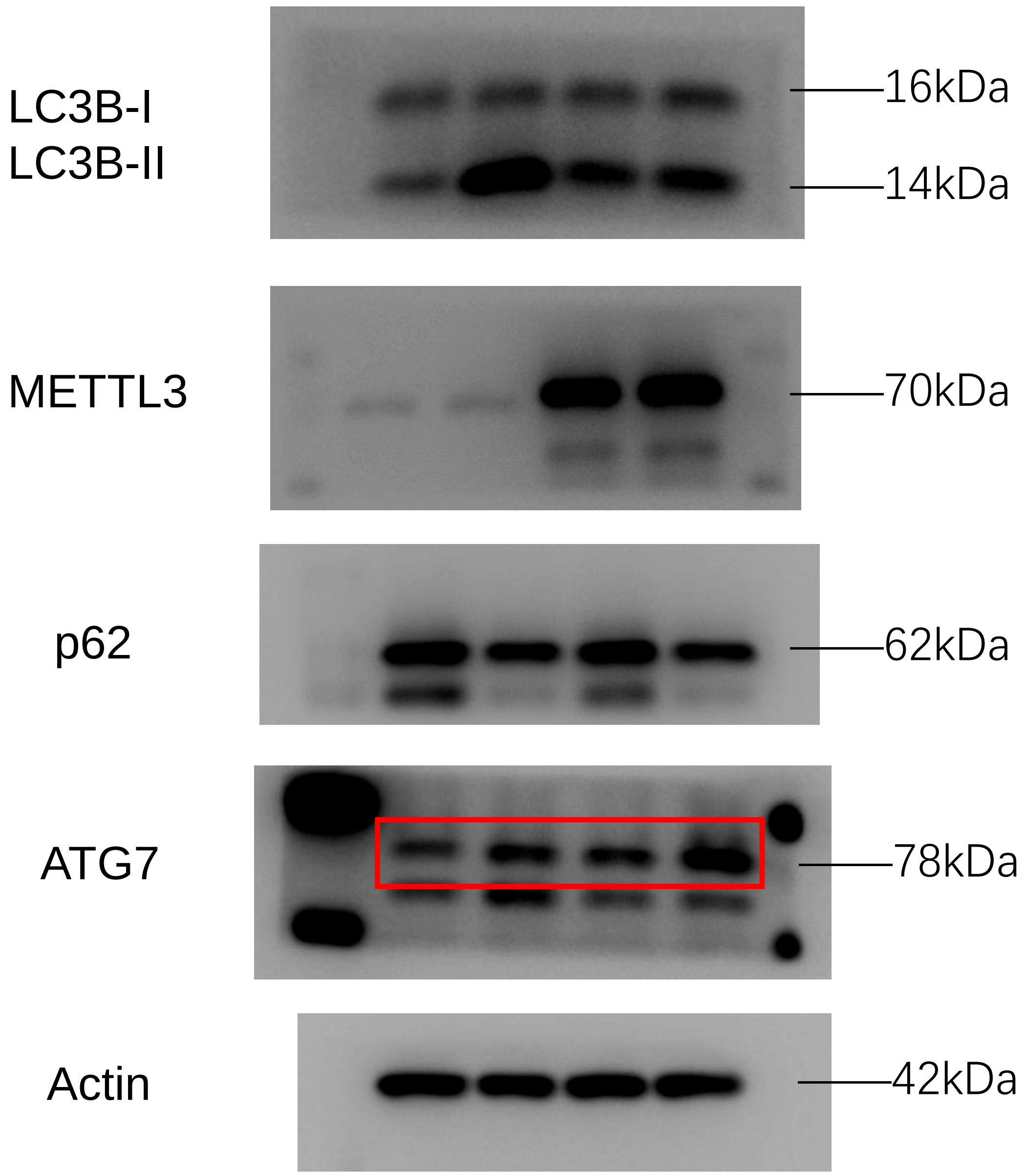

Supplementary Figure 3E

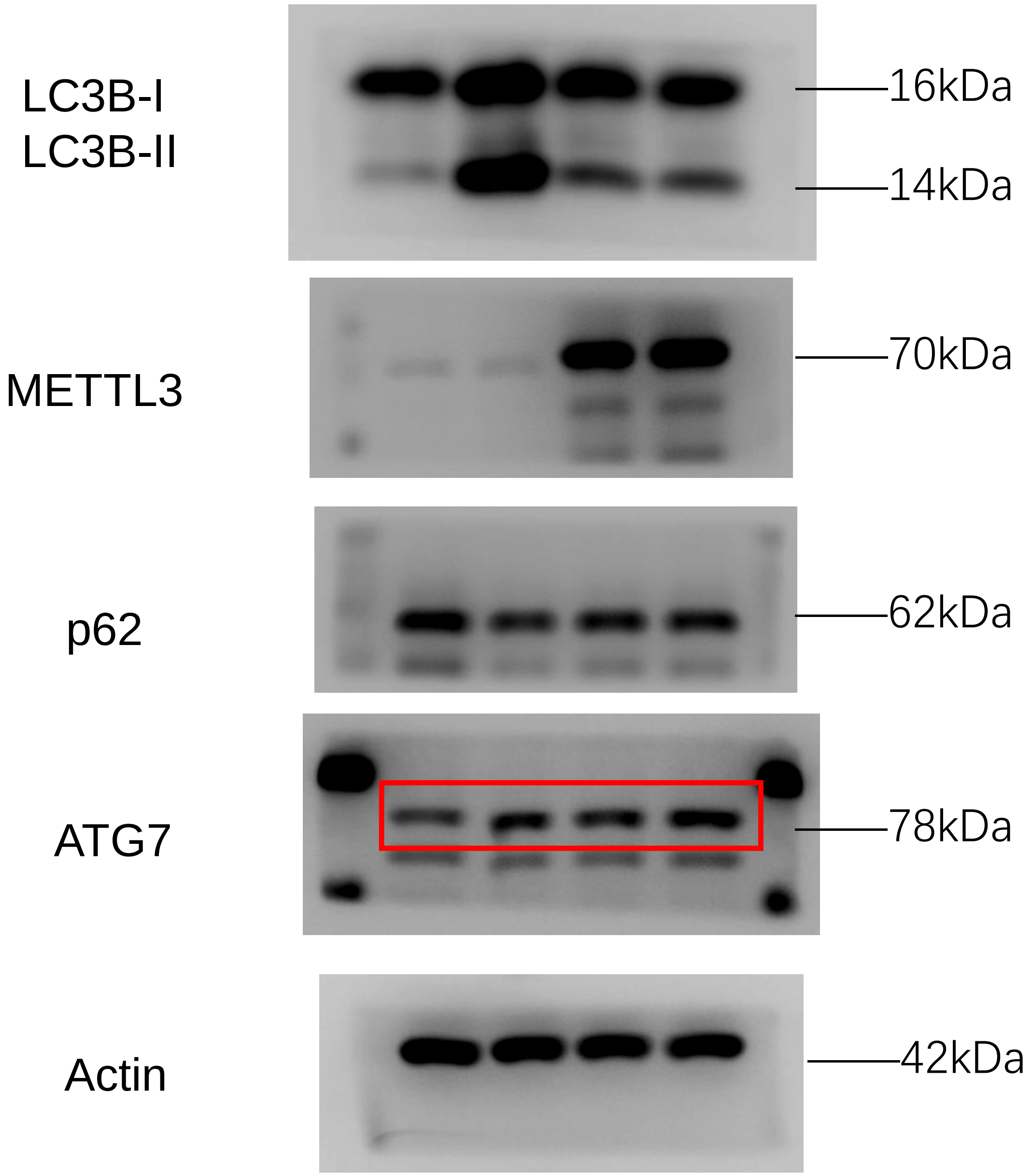

Supplement: Supplementary file 3 — Original Figures [file 41420_2025_2320_MOESM3_ESM.pdf]
